# Hydrogenation of Carbon Dioxide to Dimethyl Ether on CuO–ZnO/ZSM-5 Catalysts: Comparison of Powder and Electrospun Structures

**DOI:** 10.3390/ma16237255

**Published:** 2023-11-21

**Authors:** Aidin Nejadsalim, Hamid Reza Godini, Sanjay Ramesh Kumar, Fausto Gallucci, Delf Kober, Aleksander Gurlo, Oliver Görke

**Affiliations:** 1 Technische Universität Berlin, Faculty III—Process Sciences, Institute of Material Science and Technology, Chair of Advanced Ceramic Materials, Straße des 17. Juni 135, 10623 Berlin, Germany; aidin.nejadsalim@ceramics.tu-berlin.de (A.N.); delf.kober@ceramics.tu-berlin.de (D.K.); gurlo@ceramics.tu-berlin.de (A.G.); 2 Inorganic Membranes and Membrane Reactors, Sustainable Process Engineering, Chemical Engineering and Chemistry, Eindhoven University of Technology, 5612 AZ Eindhoven, The Netherlands; sanjayramesh014@gmail.com (S.R.K.); f.gallucci@tue.nl (F.G.)

**Keywords:** hybrid catalyst, CO_2_ hydrogenation, dimethyl ether, electrospinning, CuO–ZnO, ZSM-5, characterization

## Abstract

The promising direct dimethyl ether (DME) production through CO_2_ hydrogenation was systematically analyzed in this research by synthesizing, characterizing, and testing several catalytic structures. In doing so, various combinations of precipitation and impregnation of copper- and zinc-oxides (CuO–ZnO) over a ZSM-5 zeolite structure were applied to synthesize the hybrid catalysts capable of hydrogenating carbon dioxide to methanol and dehydrating it to DME. The resulting catalytic structures, including the co-precipitated, sequentially precipitated, and sequentially impregnated CuO–ZnO/ZSM-5 catalysts, were prepared in the form of particle and electrospun fibers with distinguished chemical and structural features. They were then characterized using XRD, BET, XPS, ICP, TGA, SEM, and FIB-SEM/EDS analyses. Their catalytic performances were also tested and analyzed in light of their observed characteristics. It was observed that it is crucial to establish relatively small-size and well-distributed zeolite crystals across a hybrid catalytic structure to secure a distinguished DME selectivity and yield. This approach, along with other observed behaviors and the involved phenomena like catalyst particles and fibers, clusters of catalyst particles, or the whole catalytic bed, were analyzed and explained. In particular, the desired characteristics of a CuO–ZnO/ZSM-5 hybrid catalyst, synthesized in a single-pot processing of the precursors of all involved catalytically active elements, were found to be promising in guiding the future efforts in tailoring an efficient catalyst for this system.

## 1. Introduction

The challenge of reducing CO_2_ emissions and the potential of utilizing renewable energy for hydrogen production can be bridged by developing an efficient catalytic technology for the hydrogenation of CO_2_ to methanol and dimethyl ether (DME) to be ultimately used as a green fuel and energy/hydrogen carrier with a lower carbon footprint [1]. DME can also be used as a solvent or further processed and converted to value-added chemicals, thereby enhancing the circular industry and economy [2,3,4]. A wide range of challenges, from technical ones, including developing an efficient catalyst and integrated process, all the way to political and regulatory ones, are still present down the road for this developing technology to become the large-scale, globally accepted, established technology [5,6]. Several attempts have been made to overcome these challenges. Recently, bifunctional catalysts have been designed and developed for the direct synthesis of DME from CO_2_ hydrogenation [7,8,9]. Among the hybrid catalysts, CuO–ZnO-solid acid catalysts with different solid acids including Al_2_O_3_, SAPO-18, and H–ZSM-5 play a significant role for this purpose [8,9,10]. In a study conducted by Krim et al., they combined a methanol synthesis catalyst of CuO–ZnO/Al_2_O_3_ with a mesoporous nano hollow ZSM-5 zeolite shell as a successive DME synthesis catalyst [11]. Hu et al. investigated the catalytic performance of several CuO–ZnO–Al_2_O_3_/HZSM-5 bifunctional catalysts with different Cu/Zn ratios and different Al_2_O_3_ dopings. The results showed that doping Al_2_O_3_ results in an increase in BET surface area, resulting in an increase in Cu distribution, which decreases the Cu crystallite size and improves catalytic performance. With a Cu:Zn ratio of 7:3, the best catalyst showed a 27% CO_2_ conversion and a 67% DME selectivity at the temperature of 260 °C under a gas flow of H_2_:CO_2_:N_2_ 3:9:1 in molar [8]. However, studies revealed that due to the hydrophilicity of Al_2_O_3_, in the catalysts consisting of Al_2_O_3_ the catalytic activity can be reduced [12,13]. To overcome this, several studies have been conducted [11,14]. Methanol is most frequently dehydrated with the H–ZSM-5 catalyst because its structure contains a high percentage of acidic Brønsted sites and is hydrophobic. Hydrophobicity makes it more resistant to poisoning caused by water [15]. A study by Xu et al. compared and tested different solid acid catalysts including Al_2_O_3_, H–ZSM-5, amorphous silica–alumina, and TiO_2_ modified by ZrO_2_. H–ZSM-5 (Si/Al = 25) showed the greatest activity among other solid acids with a DME selectivity of 20% at 280 °C among other solid acids [16]. As part of improving the activity and physicochemical properties of DME hybrid catalysts, other parameters, including the effect of doping different metal species, Cu distribution within the catalyst structure, and the size of Cu and Cu–Zn interactions, also play a crucial role [17]. A number of studies have shown that the acidity and properties of the catalyst can be changed by doping metal species such as Zr, Fe, Pd, etc. [18,19,20]. Cu dispersion can be improved by manipulating geometrical space between Cu particles since Zn plays a promoter role along with Cu to manage Cu dispersion and prevent Cu particles from sintering [17]. Depending on the catalyst synthesis/preparation parameters (e.g., Cu/Zn molar ratio), the promotion effect and Cu particle size can be different. In a study reported by Ren et al., bifunctional catalysts were synthesized by the co-precipitation method and tested toward DME synthesis. They investigated the effects of catalyst preparation and the results showed that the high activity and selectivity of DME with the value of 55.5% can be ascribed to the microstructure of the catalyst. Therefore, they showed that the activity and selectivity can be significantly affected by the catalyst preparation [12]. Nevertheless, the development of highly activated hybrid catalysts for such systems still requires systematically engineering the metal active sites to position them near the solid acids in such a way that the entire hybrid component is in optimal contact with each other. This research will focus on identifying and addressing the technical challenges in developing an efficient catalytic structure for this system, which is represented by the following main sequential reactions. The initial reaction (Reaction (1)) starts with the hydrogenation of carbon dioxide to methanol (MeOH) over metal oxide catalysts (e.g., copper–zinc oxides), followed by the dehydration of produced MeOH (Reaction (2)), which occurs over an acidic solid material such as zeolite [21].

(1) $${CO}_{2}+{3H}_{2}↔{CH}_{3}OH+H_{2}O\;\;\;∆H^{{}^{\circ}}=-49.4\;KJ\;{mol}^{-1}$$

(2) $$2{CH}_{3}OH↔{CH}_{3}{OCH}_{3}+H_{2}O\;\;\;∆H^{{}^{\circ}}=-23.4\;KJ\;{mol}^{-1}$$

There are other side reactions involved, such as reverse water gas shift (RWGS, Reaction (3)) and methanation (Reaction (4)).

(3) $${CO}_{2}+H_{2}↔CO+H_{2}O\;\;\;∆H^{{}^{\circ}}=42.1\;KJ\;{mol}^{-1}$$

(4) $${CO}_{2}+4H_{2}↔{CH}_{4}+2H_{2}O\;\;\;∆H^{{}^{\circ}}=-165\;KJ\;{mol}^{-1}$$

In particular, this research targets studying these catalytic reactions under a mild operating pressure of up to 20 bar and aims to systematically analyze the impacts of chemical–structural characteristics of various catalytic structures on their catalytic performance. The obtained knowledge can be utilized for tailoring an efficient catalytic structure for this hybrid catalytic system. In doing so, analysis tailoring of the material–chemical–structural characteristics of the benchmark CuO–ZnO and ZSM-5 catalytic materials responsible for hydrogenation and methanol dehydration catalytic reactions was performed using different synthesis methods in this research. These methods include different combinations of precipitation and impregnation of copper- and zinc-oxides over ZSM-5, which enable the establishment of different levels of distribution of the active materials for catalytic CO_2_ hydrogenation to methanol and its dehydration to DME. It has been demonstrated that the electrospinning (ES) technique is an efficient technique to produce fibers to be used in a wide range of applications such as the catalysis [22], electro- and photocatalysis [23,24], membranes [25], biomedicine [26], and magnetic refrigeration [27]. In this context, ES catalysts in the form of fibers were tested in this research along with the catalysts synthesized using the above-mentioned methods.

The desired catalytic performance in this system is materialized by obtaining a significant CO_2_ conversion to methanol while quickly dehydrogenating it to DME. This could be reached by shifting the equilibrium of reactants and products of these consecutive catalytic hydrogenation and dehydration reactions over the catalyst surface by establishing the right intensity and dynamic of activation and conversion of CO_2_ to methanol and dehydrating it to DME. Previous experimental and model-based analyses, e.g., [7,8,21,28,29], have already proven this to be the case. The applied tailoring analysis approach in this research aims to identify the targeted characteristics of the catalysts to be tailored for establishing the desired efficient catalytic performance in terms of the highest selective conversion of carbon dioxide.

The catalyst preparation activities in this research can be categorized into a) synthesizing and processing the powder-shape catalysts and b) preparing a spinnable solution followed by electrospinning it to obtain fiber-shaped catalysts. The synthesized hybrid catalysts have either the compositions of 16.7% CuO:16.7% ZnO:66.6% ZSM-5 or 33.3% CuO:33.3%ZnO:33.3% ZSM-5 (in wt.%). These, along with the synthesized references 50% CuO:50% ZnO, in wt.%, and H–ZSM-5 samples were studied in this research. 

## 2. Materials and Methods

### 2.1. Chemicals

The list and specifications of the chemicals used in synthesizing catalysts are provided in Table 1. All materials were used without further treatment/purification. All abbreviations and symbols used in this work with the corresponding expressions can be found in Appendix A in Table A1 and Table A2.

### 2.2. Catalysts Synthesis

The commercial H–ZSM-5 (CAS No. 1318-02-1) was received in the median particle size distribution of 3.4 µm and utilized without further treatment in catalyst synthesis. The synthesis procedure of the methanol catalyst is similar to the standard co-precipitation recipe reported earlier [28,30]. In this manner, as a first step in this synthesis procedure, separate aqueous solutions of copper nitrate Cu(NO_3_)_2_·3H_2_O, zinc nitrate Zn(NO_3_)_2_·6H_2_O, and sodium carbonate Na_2_CO_3_, each with a molarity of 0.2 M, were prepared. The metal nitrate solutions were mixed to establish a light blue solution with a weight ratio of 50 wt.%Cu:50 wt.%Zn. A total of 400 mL of this solution and 500–600 mL of the precipitant solution were added to 600 mL of Di-Ionized (DI) water under vigorous stirring of 500 rpm. The metal nitrates and the precipitant solutions were added with controlled drop rates for each solution. The pH of the solution was maintained around 7 ± 0.2 at the temperature range of 60–65 °C. After obtaining the precipitate, it was aged for 2 h at the same temperature and stirring conditions. After crushing and pelletizing the dried solid, it was sieved to obtain the desired size fraction of 100–250 µm. In order to remove organics and obtain the catalyst, the sieved solid was then calcined at 360 °C for 4 h with a heating rate of 2 °C·min^−1^. The obtained catalyst is referred to in this paper as a reference CP catalyst.

#### 2.2.1. Synthesis of Sequential-Precipitated Catalyst

Similar to the procedure applied for synthesizing the reference CP catalyst, aqueous solutions of copper nitrate, Cu(NO_3_)_2_·3H_2_O, and zinc nitrate, Zn(NO_3_)_2_·6H_2_O, and Na_2_CO_3_ with the molarity of 0.2 M were separately prepared. In the next step, 200 mL of Zn(NO_3_)_2_ · 6H_2_O solution and 250 mL of Na_2_CO_3_ solution were drop-wise added to DI-water containing 3.23 g of H–ZSM-5 powder and maintained at 60–65 °C and a pH of 7. The precipitate obtained from the first precipitation step was aged while stirring for 30 min. Then, 200 mL of Cu(NO_3_)_2_·3H_2_O solution was added, followed by a color change from white to light blue, showing the precipitation of the copper content. The whole precipitate was kept aging for 2 h under stirring. A pH decrease of 0.2–0.4 was observed during the second aging time. The precipitate was washed with deionized (DI) water, filtered, and dried at 80 °C overnight. After crushing and pelletizing the dried solid, it was sieved and then calcined in air atmosphere at 350 °C for 4 h with a heating rate of 2 °C·min^−1^. Finally, a catalyst with a final composition of 33.3%CuO:33.3%ZnO:33.3%H–ZSM-5, in wt.%, was obtained, which is referred to in this paper as SP catalyst.

#### 2.2.2. Synthesis of Sequential-Impregnated Catalyst

After preparing two separate 0.5 M solutions of copper-nitrate Cu(NO_3_)_2_·3H_2_O and zinc-nitrate Zn(NO_3_)_2_·6H_2_O in ethanol, 10 g of H–ZSM-5 powder was added to 40 mL of the later one, Zn(NO_3_)_2_·6H_2_O, to start the impregnation. Then, the impregnation was completed in a rotary evaporator, and the gel was dried as described in Section 2.2.1. The crushing, pelletizing, and sieving were also performed similarly. The calcination process was carried out at 300 °C for 10 h with a heating rate of 3 °C·min^−1^ and continued at 350 °C with a heating rate of 5 °C·min^−1^ for 4 h. The obtained ZnO/H–ZSM-5 was added to a 40 mL Cu(NO_3_)_2_·3H_2_O solution to perform the second impregnation sequence. A similar process was carried out for the preparation of CuO–ZnO/H–ZSM-5 as for ZnO/H–ZSM-5. The procedure was completed by calcining it at 350 °C for 4 h with a heating rate of 3 °C·min^−1^. Finally, a catalyst with a final composition of 16.7%CuO:16.7%ZnO:66.6%H–ZSM-5, in wt.%, was obtained, which is referred to in this paper as SI catalyst.

#### 2.2.3. Fluorine-Assisted Single-Pot Synthesis of CuO–ZnO/ZSM-5 (FSP)

CuO–ZnO/ZSM-5 was synthesized in a single-pot hydrothermal reactor with the assistance of fluorine as a mineralizer to facilitate the crystallization of ZSM-5, while Cu and Zn were incorporated simultaneously. In the first step of this synthesis procedure, all metal nitrates, including 5.7 mmol of Cu(NO_3_)_2_·3H_2_O, 5.7 mmol of Zn(NO_3_)_2_·6H_2_O, and 0.7 mmol of Al(NO_3_)_3_·9H_2_O were dissolved in 30 mL DI water. Subsequently, 27 mmol of NH_4_F was added to the metal nitrates solution under vigorous stirring, and then 6.64 mmol of TPAOH was added to the above-mentioned solution. Afterward, 28.3 mmol of fumed SiO_2_, as a Si source, was added to the resulting solution. The solution was then stirred for 6 h to obtain a homogeneous gel. Later, the gel was transferred into a Teflon-lined stainless steel autoclave reactor and kept at 190 °C for 8 days. After cooling it to room temperature, the wet solid was washed and centrifuged thrice at 6000 rpm for 10 min. The resulting solid was then dried at 80 °C overnight. Finally, the dried solid was calcined at 800 °C for 2 h to remove the organics and complete the crystallization process. The obtained powder is referred to as FSP-P powder catalyst in this paper and was utilized for electrospinning and catalytic testing.

#### 2.2.4. Preparation of the Spinnable Solutions

As the first step of catalyst preparation via electrospinning (ES), the spinnable solutions were prepared for all selected samples. For the co-precipitated synthesized sample, a certain amount of PVP was directly added to the metal nitrates/H–ZSM-5 solution at 40 °C under vigorous stirring. In the case of preparing the spinnable solution for the single-pot (FSP) sample, 0.4 g of FSP-P was added to 7 mL DMF, and then it was subjected to ultrasound for 1 h to disperse the FSP-P in DMF better. In the next step, PVP was added gently to the FSP-P/DMF solution under stirring. All solutions mentioned above were stirred for 18 h to obtain a homogeneous spinnable solution. The spinnable solution of commercial ZSM-5 in DMF was also prepared in the same way to ensure comparable conditions. 

#### 2.2.5. Fibrous Catalysts Fabrication

To fabricate the fiber-form catalysts, the prepared spinnable solutions, containing different metal nitrates and ZSM-5 in different ratios, were loaded in the electrospinning apparatus (Yflow^®^ 2.2.D-300, Yflow Sistemas y Desarrollos S.L., Málaga, Spain). The setup was equipped with a high-voltage power supply, a peristaltic pump, a syringe with a stainless steel needle (21 gauge), and a ground aluminum collector. After loading the spinnable solution into the syringe, it was pumped into the needle at a constant flow rate of 0.3 mL/h by the peristaltic pump. CuO–ZnO/ZSM-5/PVP fibers were obtained at the applied voltage of 15 kV and a needle-to-collector distance of 15 cm (a photo and a schematic of the electrospinning equipment are shown in Figure S1). Fibers were collected on an aluminum foil as a ground collector at ambient temperature and humidity. The collected fibers were peeled off from the aluminum foil. Then, as-spun fibers were dried at 80 °C overnight before processing them in the calcination stage. Afterward, CuO–ZnO/ZSM-5/PVP fibers were calcined at 550 °C for 4 h with a heating rate of 1 °C·min^−1^ to remove PVP and obtain metal oxides/ZSM-5 fibers. The co-precipitated CuO–ZnO/ZSM-5 fibers were labeled as CZZ (Cu-Zn-Z = ZSM-5) catalysts. CZZ (33 wt.%) and CZZ (66 wt.%) fibers were obtained corresponding to the weight percent of the zeolite in the final composition. These were utilized for further test analysis and characterization. Similarly, the obtained FSP fibers were labeled as FSP-F catalysts. Moreover, H–ZSM-5 fibers were also fabricated under the same conditions as those mentioned above to ensure a comparative study. Table 2 provides an overview of the codes and the specifications of the synthesized tested catalysts in this research.

### 2.3. Characterization

In this study, crystalline structures of the catalysts’ samples were studied using X-Ray Diffraction (XRD, Bruker D8 Advance, Karlsruhe, Germany) at room temperature with a CuKα radiation source with the wavelength of 1.54059 Å in the 2θ angles of 10° to 90°. A Rietveld refinement was conducted to obtain a more precise calculation of the phase composition and crystallite sizes of metal oxides and zeolites present in the catalysts for all powder and fiber samples. All these data were derived using TOPAS software with the released version of 4.2 (Brucker AXS GmbH, Karlsruhe, Germany). Through XPS analysis, the samples’ electronic structure and surface chemistry were studied, for instance, to determine the oxidation states of the metal species and the composition of the metals on the surface. All synthesized samples including the methanol reference CP catalyst; hybrid catalysts of SP, SI, CZZ(33), and CZZ(66); and fluorine-assisted single-pot (FSP-P) were analyzed by XPS after the calcination step. For a better comparison of all metal active site species present in the samples (e.g., Cu and Zn), Cu(NO_3_)_2_·3H_2_O, and Zn(NO_3_)2·6H_2_O powders were calcined at 550 for 4 h to obtain pure CuO and ZnO, labeled as CuRef and ZnRef, respectively. The XPS analysis was conducted with the THERMOSCIENTIFIC K Alpha source gun (Thermo Fischer Scientific, Waltham, MA, USA), with a spot size of 400 μm, an energy step size of 0.1 eV, and energy steps of 601. The XPS curves were all fitted using Origin 2018^®^. Energy correction was performed for all XPS spectra based on the 284.8 eV binding energy (BE) of C1s. The surface area and pore size were measured for the synthesized samples in the powder form including CP, SP, SI, FSP-P, as well as in the form of electrospun fibers consisting of CZZ(33), CZZ(66), and FSP-F. The commercial H–ZSM-5 in powder (H–ZSM-5-P) and fiber shapes (H–ZSM-5-F) were also analyzed to better investigate the effect of electrospinning and incorporation of metal active sites on the surface area. Samples were first loaded in quartz tubes with stem and bulb, then they were degassed under vacuum at 150 °C for 12 h in an outgassing station. In the next step, the surface area of the outgassed samples was measured based on Brunauer–Emmett–Teller (BET) theory using N_2_ adsorption–desorption at a cryogenic temperature of 77 K (Quantachrome, QuadraSorb SI, Boynton Beach, FL, USA). Data acquisition and reduction were conducted for all data using the software of Quantachrome QuadraWinTM with a released version of 5.05. External surface area and microporous volume were derived from the t-plot method [31]. Total pore volume was calculated at the relative pressure of near unity (${P/P}_{0}\approx 0.99$). Mesoporous volume was obtained by subtracting microporous volume from the total pore volume. Pore diameter was derived using the Barrett–Joyner–Halenda (BJH) method. The microstructural features and morphology of the synthesized powder and electrospun fiber samples were studied using scanning electron microscopy (SEM, Leo Gemini 1530, Zeiss, Jena, Germany). The elemental analysis was performed using energy-dispersive X-ray spectroscopy (EDS). The samples were prepared for this by scattering a carbon layer on top of each sample to inhibit the charging during the characterization. Imaging was carried out in three different magnifications of 5 k×, 10 k×, and 20 k× by applying an accelerating voltage of 5 kV and adjusting the aperture size at 30 µm with a working distance of 11 to 13 mm. In order to investigate the metal active sites distribution through the zeolite crystals, a further analysis was conducted on reference CP catalyst and the hybrid catalysts including SP, SI, and FSP-P through the combination of SEM and a focused ion beam scanning electron microscopy (FIB-SEM). FIB-SEM was conducted using a ZEISS Crossbeam 340 (Carl Zeiss Microscopy GmbH, Jena, Germany) at the HZB CoreLab Correlative Microscopy and Spectroscopy (CCMS)). A gallium ion beam was sputtered on the target area of the sample using a gallium gun. Sample preparation was performed similar to the SEM analysis (Section 2.2.4). Images have been detected using an angled detector type of Xflash 6|100 with a window type of slew AP3.3 and a take-off angle of 35° (Esprit 2.x, Bruker Nano GmbH, Berlin, Germany). The composition of all catalysts was analyzed by inductively coupled plasma optical emission spectroscopy (ICP-OES) technique using a Horiba Scientific ICP Ultima2 (Horiba, Kyoto, Japan). Prior to analysis, 0.01 g of each sample was added to an acid solution of 60% nitric acid in de-ionized water and stirred for 24 h at 80 °C to completely dissolve all compounds. Then, 500 μL of the above solution was diluted in a 5% nitric acid aqueous solution to obtain a 25 mL sample solution for measurement. To analyze the weight loss and gain and the calcination temperature of the single-pot synthesized catalyst, a thermal gravimetric analysis was performed at a heating rate of 10 °C min^−1^ with STA 409 PC LUXX (Netzsch, Selb, Germany) coupled with a mass spectrometer OMNi Star GSD 320 (Pfeiffer Vacuum, Asslar, Germany).

### 2.4. Testing the Catalytic Activity

#### 2.4.1. Description of the Reactor Setup

The catalysts were tested in a 4-fold parallel packed-bed reactor setup with an inner diameter of 9.4 mm and a length of 645 mm. The operating temperature of the catalyst bed was monitored by thermocouples at three points along the catalyst bed. The catalyst was placed inside the isothermal zone of the reactor, which is 12.5 cm in length. The gas dosing unit consists of mass flow controllers (MFC) for H_2_, CO_2_, N_2_, and Ar as an internal standard. The effluent gases were diluted with a low-pressure N_2_ gas to prevent condensation. The diluted effluent gas was analyzed with a gas chromatograph (GC, Agilent 7890B) after reactor selection with a multiposition valve heated at 180 °C. The gas chromatograph was equipped with a Polyarc system, oxidizing every C-containing effluent from the column to CO_2_, and following reduction to CH_4_. This allows the detection of CO and CO_2_ with the more sensitive FID detector and easy calibration and later quantification of all oxygenates. Permanent gases were separated on an HP-Plot 5A Molsieve column (30 m × 0.53 mm × 50 µm) together with a TCD detector. CO_2_ and all organic compounds up to C6 alkanes and C4 oxygenates are separated on a Poraplot Q column (30 m × 0.53 m × 40 µm, Agilent) coupled to a FID detector.

#### 2.4.2. Criteria and the Performance Indicators for Catalytic CO_2_ Hydrogenation to DME

Targeting the highest selectivity and yield of DME is the key practical aspect to be considered in identifying the criteria based on which the performance of such hybrid catalysts can be quantified and compared. It should be also taken into account that this usually, but not always, overlaps with another criterion to secure a significant amount of converted CO_2_ in the form of both methanol and ultimately DME (the most preferred species) in this set of consecutive Reactions (1) and (2). The CO_2_ conversion (X) and the selectivity towards different product species (Si, i = CO_2_ MeOH, and DME) were calculated using the following equations:

(5) $$X_{{CO}_{2}}=\frac{F_{CO}+F_{{CH}_{4}}{+F}_{{CH}_{3}OH}+2F_{C_{2}H_{6}O}}{F_{{CO}_{2,}\in }}\times 100$$

(6) $$S_{MeOH}=\frac{F_{{CH}_{3}OH}}{F_{CO}+F_{{CH}_{4}}{+F}_{{CH}_{3}OH}+2F_{C_{2}H_{6}O}}\times 100$$

(7) $$S_{CO}=\frac{F_{CO}}{F_{CO}+F_{{CH}_{4}}{+F}_{{CH}_{3}OH}+2F_{C_{2}H_{6}O}}\times 100$$

(8) $$S_{DME}=\frac{{2F}_{C_{2}H_{6}O}}{F_{CO}+F_{{CH}_{4}}{+F}_{{CH}_{3}OH}+2F_{C_{2}H_{6}O}}\times 100$$

(9) $$Y_{DME}=\frac{X_{{CO}_{2}}\times S_{C_{2}H_{6}O}}{100}$$

(10) $$Y_{MeOH}=\frac{X_{{CO}_{2}}\times S_{{CH}_{3}OH}}{100}$$

where ∈ indicates the number of moles of CO_2_ entering the reactor and F is attributed to the mole of each specie in the reactor’s outlet gas stream. The multiplication of its selectivity to the CO_2_ conversion in this case indicates the yield of each product species. Having considered these, maximizing the yield of the desired products (DME and methanol), primarily the DME yield, is one of the main performance indicators of catalytic testing of the prepared samples. Nevertheless, it is difficult to always secure a high level of CO_2_ conversion for all catalytic samples and then compare the products’ yield. Therefore, comparing the selectivity of species even at low but comparable levels of CO_2_ conversion also indicates the selective behavior of each catalytic sample.

#### 2.4.3. Design of Catalytic Tests

The catalytic activity testing was carried out for all catalysts and catalytic structures, including the co/sequential precipitation/impregnation, single-pot, and the corresponding electrospun fibrous catalysts. The performance of all catalysts was evaluated by setting the operating pressure values at 10 bar or 20 bar, operating temperature at 200 °C, 230 °C, or 260 °C, and GHSV in the range of 4600–18,200 mL·h^−1^·g^−1^. In this manner, 250 mg of each catalyst first was diluted with 2250 mg of SiC and then loaded into the reactor. Table 3 represents the design of the experiment for the catalytic performance test of each catalytic sample conducted in this study. The activity test was designed in a 9-step procedure. The catalysts were initially reduced at 300 °C for 2 h with a heating rate of 10 °C·min^−1^, under atmospheric pressure, while feeding a gas mixture of 50%H_2_–50%N_2_ with a flow rate of 40 mL min^−1^. After completion of the reduction step, the catalysts were cooled down to 260 °C under N_2_ flowing by a flow rate of 40 mL.min^−1^. Then, a reaction mixture of the molar ratio of 3H_2_:1CO_2_ and the exact composition 21%CO_2_:64%H_2_:15%N_2_ was fed to the reactor with the specified flow rates for each step reported in Table 3. The pressure was then increased to the designed value of 20 bar. One experiment (Step 3 in Table 3) was repeated as a reproducibility check at the temperature of 260 °C and GHSV of 4600 mL·h^−1^·g^−1^. To investigate the influence of GHSV on the performance, three different values of GHSVs, 4600 mL·h^−1^·g^−1^, 9200 mL·h^−1^·g^−1^, and 18,200 mL·h^−1^·g^−1^ were established at the constant temperature 230 °C and operating pressure of 20 bar. The pressure then was reduced to 10 bar at the same temperature of 230 °C and GHSV of 4600 mL·h^−1^·g^−1^ followed by a repeat test at the pressure of 20 bar (Table 3, Steps 1–3). The reactor test was performed at other temperatures (230 and 200) and pressures (10 and 20 bar) for each of the operating conditions found in Table 3. The performance of different catalysts could be compared under the same set of operating conditions. In this manner, the effects of each parameter (i.e., GHSV, temperature, and pressure) can be investigated while the other parameters are kept constant.

## 3. Results and Discussion

The results of the performed characterizations, followed by the observed performances of the catalytic testing, are reported in this section. These data are discussed in the framework of identification and possible correlation of the characteristics of the catalysts’ samples with their catalytic performances.

### 3.1. Characterization Results

**Figure 1:**
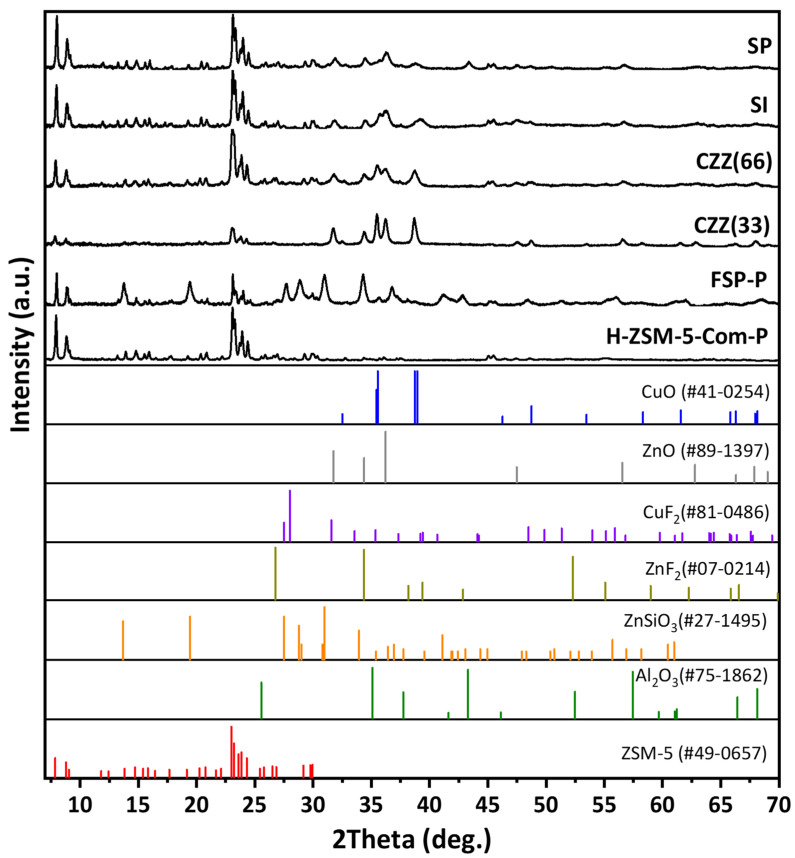
XRD patterns of all investigated catalytic samples.

All samples were analyzed using XRD to investigate the effect of introducing Cu and Zn by different methods on the crystalline structure of the zeolite (ZSM-5). In particular, the material phase characteristics of the hybrid synthesized single-pot (FSP) catalyst were studied. Additionally, all samples’ crystalline structures and phases were compared to the parent H–ZSM-5 (commercial). Figure 1 shows the XRD patterns of all samples, where for all hybrid catalyst samples the feature reflections corresponding to MFI (ZSM-5) can be found at 2θ = 21–25°, according to the data bank JCPDS No. 49-0657. All hybrid samples exhibit the corresponding reflections of CuO (JCPDS No. 41-01254) and ZnO (JCPDS No. 89-1397). The peak assigned for CuO at 2θ of 35.5° is present in all samples, except H–ZSM-5. However, it is sharper in CZZ(33), implying the presence of larger CuO crystallites in this sample, as seen in Figure 1. Meanwhile, CZZ(66) shows higher ZSM-5 intensity than CZZ(33) due to its twice higher ZSM-5 content (66%). As the metal content increased in CZZ(33), the corresponding Cu and Zn reflections became sharper and narrower in comparison to their counterparts (CZZ(66)). As can be seen for CZZ(33), CZZ(66), and FSP, the 35.43° reflection corresponds to (002) crystal planes inside CuO. Figure S2 shows the XRD patterns corresponding to acidic ZSM-5 and the unsuccessful attempt to synthesize ZSM-5 without fluorine in the presence of Cu and Zn nitrates. As a result of the experiment, acidic ZSM-5 was obtained. However, the introduction of Cu and Zn metal nitrates under the same hydrothermal conditions did not result in the expected ZSM-5 feature reflections (Figure S2). Upon introducing fluorine under the selected conditions, ZSM-5 was able to be crystallized in FSP-P (Figure 1). The FSP-P sample presents an additional composition of zinc metasilicate (ZnSiO_3_) that might be attributed to the inter-fusion between ZnO and SiO_2_ during the heat treatment at higher temperatures [32]. In order to investigate the phase transformations and determine the calcination temperature of the as-synthesized FSP sample, a TGA analysis was performed, which covered the temperature range of room temperature to 1100 °C, as can be seen in Figure S3. Moreover, the XRD patterns of FSP at different calcination temperatures were plotted in Figure S4. As seen, increasing the calcination temperature decreased the crystallinity of the planes found in ZSM-5. There are reflections associated with ZnSiO_3_ in FSP (Figure 1), possibly as a result of the formation of a solid solution between Zn and Si during the calcination step at above 600 °C, as shown in Figure S4 where the matched reflection appeared at 650 °C. The catalytic materials showing the highest zeolite crystallinity (i.e., SI, SP) are expected to be more active for methanol dehydration. This was confirmed by analyzing their catalytic performance indicators, primarily through their DME selectivity.

**Figure 2:**
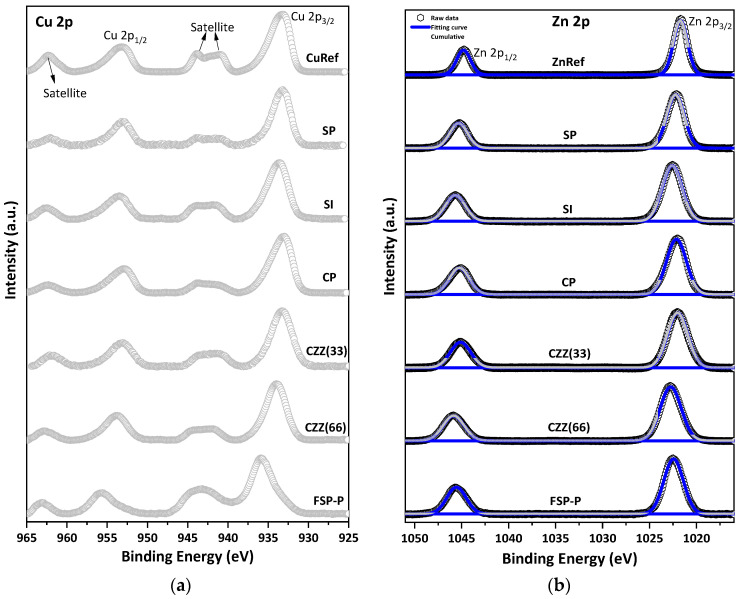
XPS spectra of (**a**) Cu 2p, and (**b**) Zn 2p for all samples. CuO and ZnO were used in a pure form as reference materials.

The chemical states of metal species (e.g., Cu) over the catalysts’ samples were studied using XPS characterization. Depending on the applied synthesis method, i.e., precipitation, impregnation, and single-pot synthesis, different levels of dispersion of the metal species [28] and quantities of active sites over the catalyst surface can be expected. An XPS measurement was performed to reveal the electronic and surface chemical states of the involved metal species. This is visualized through a comparative XPS analysis of the catalysts synthesized via sequential, co-precipitation/impregnation, electrospinning, and single-pot syntheses. Figure 2a illustrates the XPS spectra of Cu 2p plotted from 925 eV to 965 eV for all studied samples. The Cu 2p spectra mainly show four sub-peaks, including doublets of Cu 2p_1/2_ and Cu 2p_3/2_ with the spin-orbit splitting of about 19.8 eV and two shakeup satellite peaks [33]. In order to better understand the chemical shifts in all samples, they were compared with the XPS spectra of pure CuO and ZnO, labeled as CuRef and ZnRef, respectively. It has been reported elsewhere that the shakeup satellite peaks appear at 5–10 eV below the principle line in Cu 2p spectra [34], whereas such peaks cannot be found in Zn 2p spectra. These peaks particularly represent the presence of Cu(II). Having considered this and as seen in Figure 2a, the two shakeup satellite peaks located at about 943 eV and 963 eV show the presence of the paramagnetic chemical state of Cu^2+^ in all samples (Figure 2a) [35]. The doublets of Cu 2p_3/2_ and Cu 2p_1/2_ at 933.73 eV and 953.27 eV can be clearly seen in Figure 2a for the CuRef sample. The feature peaks at 933.32 eV and 953.22 eV are observed for the SP catalyst with a chemical shift of 0.05 eV to the lower BEs. The same doublet peaks can be observed for the SI catalyst at 934.34 eV and 954.24 eV, while a shift towards higher BEs can be seen for this catalyst compared to SP. In addition, the shakeup satellite peaks of the SI catalyst are more intense than those of the SP catalyst, which can be correlated to the existence of Cu metallic in SP. For the CP catalyst, the feature Cu 2p peaks are at 933.7 eV and 953.45 eV, similarly attributed to Cu 2p_3/2_ and Cu 2p_1/2_, respectively. As can be seen from Figure 2a, the catalysts prepared by the sequential impregnation method, namely SI catalyst, exhibit a higher shift compared to the corresponding spectra (Cu 2p and Zn 2p) in the catalysts prepared by sequential/co-precipitation approach, namely SP and CP catalysts. For electrospun fiber catalysts, including CZZ(33) and CZZ(66), all characteristic peaks of Cu 2p_3/2_ and Cu 2p_1/2_ can be observed at 933.95 eV and 953.7 eV. Moreover, shakeup satellite peaks are present in the corresponding spectra. A slight shift to higher BE can be seen for CZZ(66), which might be due to the lower amount of metal active sites present in this catalyst compared to the CZZ(33) catalyst. As can be seen for FSP-P XPS in Figure 2a, it exhibits a triangular line shape, while the other samples display nearly square line shapes. One of the satellite peaks in FSP-P disappeared along with the main peak shifting to the higher BEs of about 936.52 eV. This XPS pattern and shift to higher BEs can be attributed to CuF_2_ [34,36,37]. This results from the solid-state effect of hybridization between 3d and ligand orbitals in the FSP-P catalyst [36,37,38], in which NH_4_F was used as a mineralizer so that Cu reacted with F in the synthesis medium of the single-pot.

**Figure 3:**
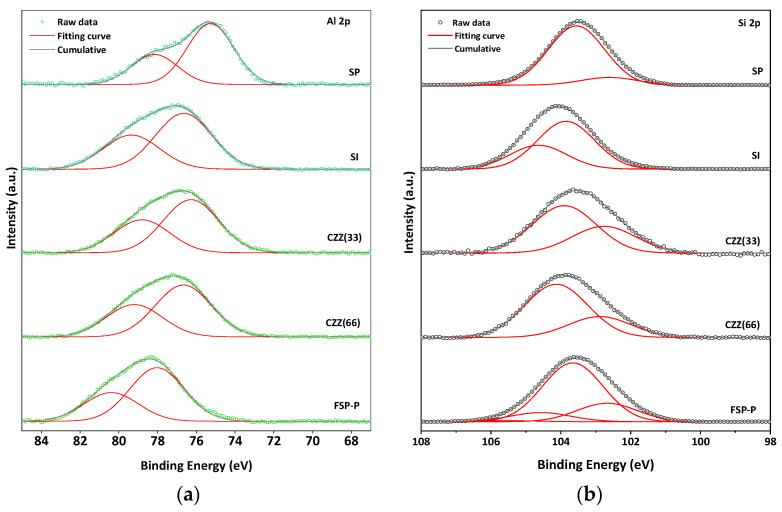
XPS spectra of (**a**) Al 2p and (**b**) Si 2p.

Figure S5 shows the XPS of C 1s for all samples. As can be seen in the C 1s spectra, a different FWHM can be observed for all samples, meaning the different ratio of carbon is available for each sample. The BE shifts can be attributed to the charge transfer that occurred in all samples [38]. In the Zn 2p XPS spectra, Figure 2b, the characteristic doublet peaks attributed to Zn 2p_3/2_ and Zn 2p_1/2_ can be observed in all catalyst samples. The gap between the prominent doublet peaks in Zn 2p XPS is about 23 eV [39]. For the SP catalyst, the peaks at 1022.1 eV and 1045.3 eV can be ascribed for Zn 2p_3/2_ and Zn 2p_1/2_, respectively. The synthesis and preparation methods can highly affect the surface chemistry states of the metal contents, especially the Zn species [40]. There is a shift to the higher BE for SI compared to SP. As reported elsewhere, the Zn species located inside the cation exchange sites in the zeolite structures possess higher BE because the electronegativity of lattice oxygen in zeolite is higher than the O^2−^ found in ZnO [40], stabilized on protonic acid sites, a blue shift can occur in such catalysts [41], as seen in Figure 2b. All deconvoluted feature peaks along with the corresponding BE shifts for Cu 2p and Zn 2p are shown in Tables S1 and S2, respectively. Figure 3 shows the results of the XPS analysis of Si 2p and Al 2p for all catalysts. The XPS data corresponding to Al 2p clearly show the presence of higher Al oxidation states in all samples, further confirmed by the presence of an Al–O reference peak at 75 eV, indicating the presence of Al–O bonds. Al 2p spectra of SP show two main peaks corresponding to aluminum compounds at BEs of 75.2 eV and 78.2 eV, which can be attributed to Al_2_O_3_ and anhydride Al_2_O_3_, respectively [42,43,44]. A shift to the higher BEs appeared in the SI catalyst’s spectra, which can be attributed to the higher amount of zeolite present in this catalyst (66.7%wt). Since the same amount of ZSM-5 present in the SI catalyst was utilized in CZZ(66), it showed a similar pattern shifting to the higher BEs compared to CZZ(33). It confirms the higher Al oxidation states in CZZ(66). As compared to other samples, FSP exhibited a significantly higher chemical shift. The chemical shift to the higher BE indicates the possibility of the reaction of some Al with F^−^ to make AlF_3_ with the corresponding peak of 78 eV [45]. Nevertheless, it is still difficult to conclude that Al is only bound to F^−^, as also zeolite was obtained for this sample, according to the XRD pattern (Figure 1), meaning that Al participated in the zeolite framework as well. Figure 3b represents the XPS spectra for Si 2p for all samples. Two main peaks expected by the presence of Si 2p are located at 101.4 eV and 102.4 eV corresponding to Si–O–Al and Si–O–Si compounds, respectively. The chemical shift to the higher BEs can be observed for all samples. SP exhibits the peaks mentioned above at 102.6 eV and 103.5 eV [46]. XPS analysis was also performed for the spent catalysts and compared with the fresh ones, as shown in Figure S6.

Table 4 shows the phase composition and average crystallite sizes of ZSM-5 and CuO for powder and fiber samples. The data were derived using XRD Rietveld refinement and ICP analysis. The phase compositions derived from XRD confirm the presence of the ZSM-5, CuO, and ZnO compounds in all hybrid powder and fiber samples, which are comparable with the intended compositions, in wt.% The Cu/Zn ratio was calculated based on ICP and XPS measurements, which are about 1 for all samples. As seen, SI and SP possess the smallest CuO crystallites with 7 nm and 8 nm, respectively, compared to the other samples.

**Figure 4:**
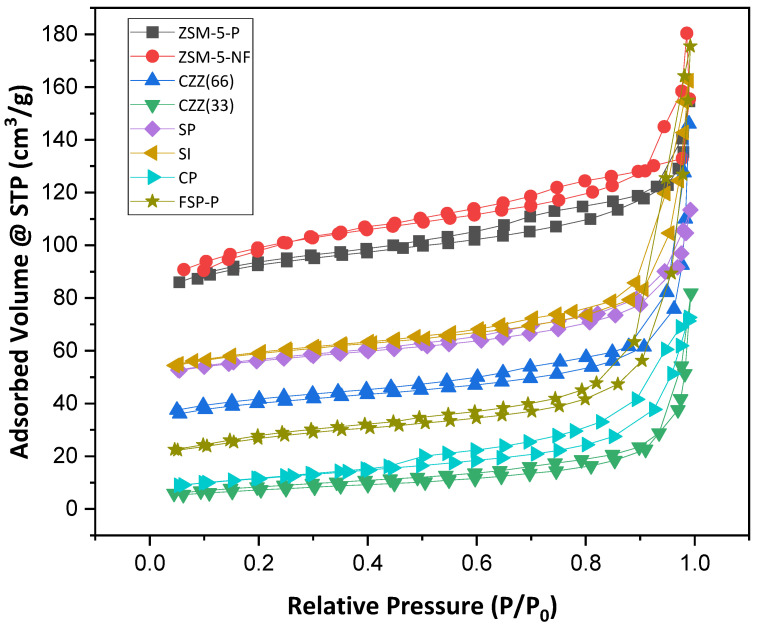
Nitrogen adsorption–desorption isotherm of all samples at 77 K, including ZSM-5-P, ZSM-5-F, CZZ(33), CZZ(66), SP, SI, CP, and FSP-P.

Figure 4 presents the Nitrogen adsorption–desorption isotherm performed at 77 K for all samples. As seen there, ZSM-5-P and ZSM-5-F show isotherm Type-I with hysteresis meaning that microporosity exists in both samples. A surface area of 294 m^2^·g^−1^ was obtained for the ZSM-5-P sample, while after the ES process, a higher surface area of 326 m^2^·g^−1^ was achieved for ZSM-5-F, confirming that fibers exhibit a higher surface area. Table 4 provides a comparative overview of such data for all catalysts’ samples. Similar isotherm types were obtained for CZZ(33) and CZZ(66) catalysts. However, introducing Cu and Zn nitrates to ZSM-5 in CZZ(33) and CZZ(66) fiber catalysts reduced the surface area significantly in these samples to 26 and 132 m^2^·g^−1^, respectively. The trends of CP and CZZ(33) catalysts are similar. However, the CP catalyst has demonstrated a slightly higher surface area of 41 m^2^·g^−1^, but both show a low portion of microporosity. This can be attributed to the higher amount of CuO and ZnO (each one 33%); through their precipitation, the access to the zeolite structure is limited, for instance, in the CZZ(33) catalyst. In contrast, CZZ(66), with more zeolite and lower metal contents, shows almost five times higher surface area than the CZZ(33) catalyst. All two SP and SI catalysts exhibit relatively similar ranges of surface areas of 183 m^2^·g^−1^ and 192 m^2^·g^−1^, respectively. Therefore, exhibiting a higher surface area by pure zeolites than the sequential/co-precipitation and impregnated catalytic samples can be attributed to the free protonic sites and channels found in pure zeolite [47]. Accordingly, by introducing metal contents in the zeolite structure, these sites and pores get blocked, causing the reduction in the surface area [47]. The catalyst synthesized by single-pot (FSP) revealed a surface area of 91.8 m^2^·g^−1^, which is slightly lower than what was obtained for SP and SI catalysts but higher than the CP catalyst. Despite the lower surface area for the FSP-P catalyst, it showed a higher mesoporous volume than the other samples.

Reviewing the recorded values of the external- and micropore surface areas for each sample and their average pore size provides valuable information regarding the desired structural characteristics of such hybrid catalysts. As seen through the data reported in Table 5, introducing zeolite has caused a reduction in the pore size to below 2 nm. They may trap some of the water molecules in the micropores and accordingly hinder the accessibility of the active sites for the reactants. Electrospinning the samples increases the relative ratio of the external surface area to the micropores’ internal surface area; therefore, a higher conversion could be expected. The CP catalyst consists of CuO and ZnO and can be considered as a reference catalyst, also in this regard representing a low but externally accessible surface structure facilitating a high level of CO_2_ hydrogenation. The CZZ(33), consisting of a significant part of the CuO–ZnO material structure, also shows a relatively low specific surface area. The precipitated metal oxides could have even blocked access to the zeolite porous structure, indicated by the recorded low micropore surface area in this hybrid catalyst. It is noteworthy to mention that the surface area of fibers can potentially be increased by optimizing the solution parameters such as precursor concentration, polymer selection, and operating parameters such as needle-to-collector distance, flow rate, and the applied voltage. Moreover, surface acidity of all catalysts was investigated and compared with the one measured for H–ZSM-5-P using temperature programmed desorption of ammonia (NH_3_–TPD), as shown in Figure S7.

**Figure 5:**
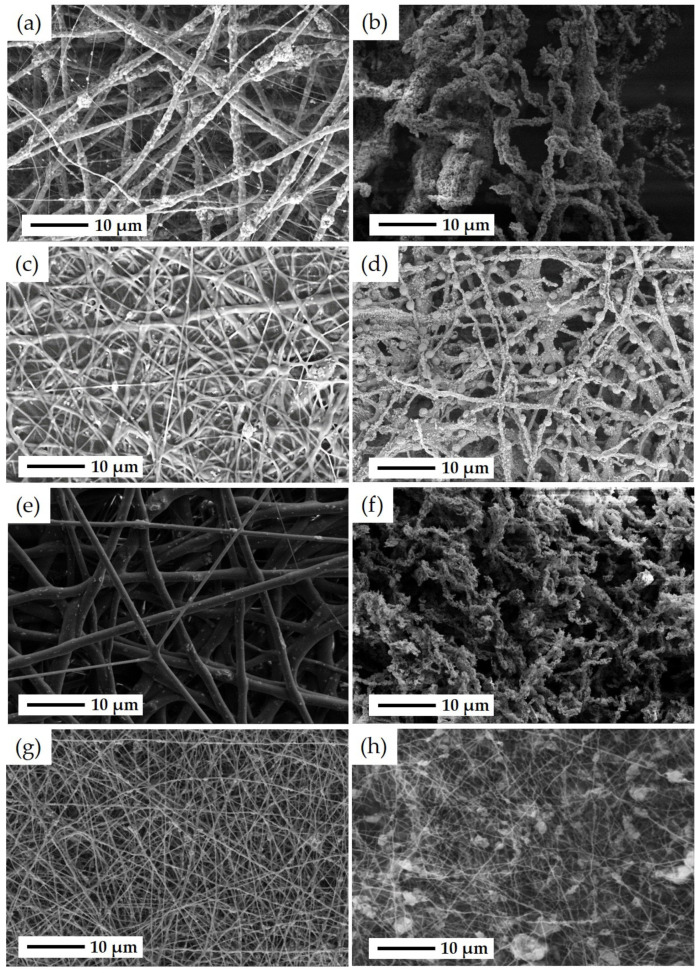
SEM images of (**a**) as-spun PVP/ZSM-5 fibers after ES and before the calcination step, (**b**) ZSM-5 fibers obtained after the calcination step and removing PVP from the fiber structure after which ZSM-5-Fs were distorted, (**c**) as-spun PVP/CZZ(33) fibers after ES and before the calcination step, (**d**) CZZ(33) fibers after the calcination step and removing PVP from fiber structure in which some spherical CuO particles can be found, (**e**) as-spun PVP/FSP fibers with a smooth structure after ES and before the calcination step, (**f**) FSP-F with shortened and distorted network after the calcination step and removing PVP from the fiber structure, (**g**) as-spun PVP/CZZ(66) fibers after the ES process and before the calcination step, and (**h**) CZZ(66) fibers after the calcination step and removing PVP.

SEM and EDS analyses were performed to investigate the microstructure and fine distribution of metal content in the catalysts synthesized by different methods. Figure 5 illustrates SEM images of the electrospun fibrous samples, including ZSM-5-F, CZZ(33), FSP-F, and CZZ(66), before and after removing the PVP during the calcination step. Figure 5a shows the as-spun PVP/ZSM-5-F morphology before the calcination step, illustrating a nonwoven network of PVP/ZSM-5 fibers with a diameter of 1.5 μm to 2 μm. As seen for this sample, in some regions, the ZSM-5 particles protruded from the surface of the fiber, which can be attributed to the aggregation of zeolite particles during the electrospinning process. Furthermore, some areas exhibit PVP-rich fibers with a diameter of about 100 nm having a few zeolite particles inside. As shown in Figure 5b, the diameter of fibers was not significantly changed for ZSM-5-F after the calcination process at 550 °C. However, as seen in Figure 5b, the fibers were distorted after removing PVP during the calcination process and a few ZSM-5-Fs were aggregated along their length. The microstructure of as-spun PVP/CZZ(33) fibers can be seen in Figure 5c. A rough fiber mat was obtained for PVP/CZZ(33) with a range of fiber diameters from 500 nm to 2 μm. There is no significant change in the diameter of fibers after the calcination step in CZZ(33) (Figure 5d). However, some spherical particles of approximately 2 μm appeared in this sample after the calcination step, which can be attributed to their copper and zinc metal contents. To investigate the spherical particles found through the CZZ(33) fibers, EDS analysis was performed (Figure S8). As seen in Figure S8, the spherical particles can be attributed to Cu. Figure 5e presents the SEM image of as-spun PVP/FSP fibers before calcination with a diameter range from 1 μm to 2.5 μm. As seen in Figure 5e, a smooth fiber structure was obtained for the PVP/FSP sample. However, after the calcination step and removing the PVP, the morphology of the fibers for the FSP-F sample was changed to a discontinuous network, and in a similar way to ZSM-5 fibers they were distorted after removing PVP. As seen in Figure 5f, the diameter of fibers for FSP-F was reduced from 500 nm to 2 μm. As-spun PVP/CZZ(66) is shown in Figure 5g, in which continuous fibers with a diameter of 500 nm can be observed, while some regions exhibit an aggregation of ZSM-5 particles in the base of fibers. The reduction in diameter can be attributed to the metal concentrations that are lower in CZZ(66) (with 16 wt.% CuO and 16 wt.% ZnO) than in CZZ(33) (with 33 wt.% CuO and 33 wt.% ZnO) caused by obtaining more continuous fibers of ZSM-5/CuO–ZnO in CZZ(66) (Figure 5h). Some agglomerated ZSM-5 powder can be found in some areas in CZZ(66) after the calcination step.

**Figure 6:**
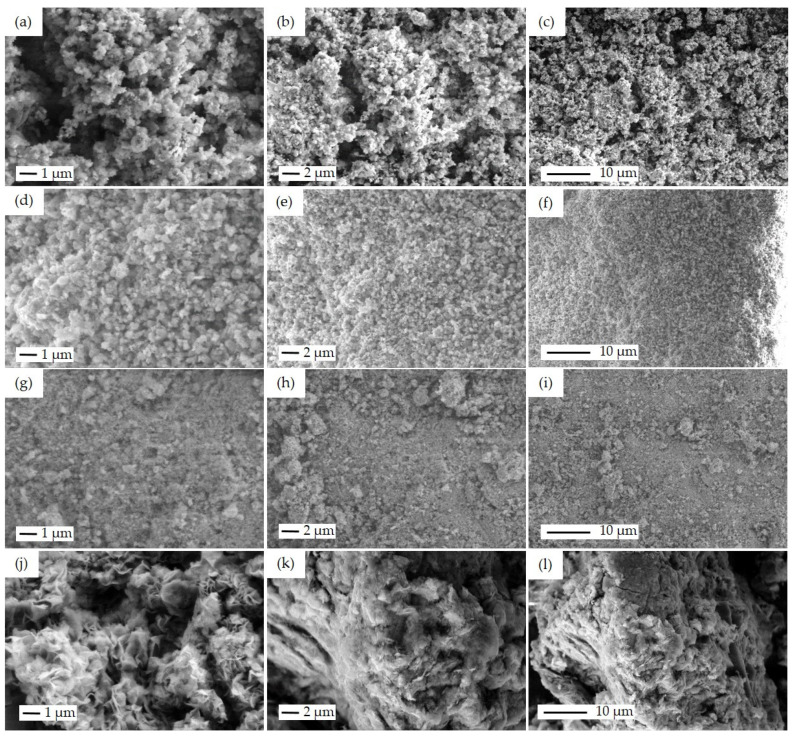
SEM images of the powder catalysts: SP with the particle size of ZSM-5 about 500 nm in different magnifications of (**a**) 20 k×, (**b**) 10 k×, (**c**) 5 k×, SI with the particle size of ZSM-5 about 500 nm in different magnifications of (**d**) 20 k×, (**e**) 10 k×, (**f**) 5 k×, CP with the magnifications of (**g**) 20 k×, (**h**) 10 k×, (**i**) 5 k×, and FSP-P with the magnifications of (**j**) 20 k×, (**k**) 10 k×, (**l**) 5 k×.

The rest of the samples with particle morphology were also investigated by the SEM technique. Figure 6 shows SEM images of the non-fibrous samples, including SP, SI, CP, and FSP-P in different magnifications of 20 k×, 10 k×, and 5 k×. For SP (Figure 6a–c), the combination of CuO, ZnO, and ZSM-5 crystallites can be observed. CuO and ZnO have been distributed around ZSM-5 crystallites, yet, in some areas, metal oxides have become agglomerated. As a result, a precise estimation of metal oxide particle size is difficult. The particle size of ZSM-5 is in the range of 500 nm to 1 μm. For the SI catalyst, fine distribution of CuO and ZnO particles across the zeolite structure could be observed, as shown in Figure 6d–f. As the CuO precursor was sequentially impregnated after impregnating the ZnO precursor on the zeolite particles in SI, it is expected to observe the CuO particles on the surface of this catalyst. Similar to the SP catalyst, the aggregation of particles can also be seen for the SI catalyst, showing the tendency of metal oxide particles to aggregate. Figure 6g–i is the SEM images of the CP catalyst. More extensive aggregation of metal oxides in some areas occurred while the CP catalyst was synthesized through the co-precipitation method simultaneously using two metal precursors. FSP-P catalyst morphology differs markedly from others, as seen in Figure 6j–l. As reported elsewhere [48], CuO/ZnO can emerge in petal-like morphologies. So, it can be concluded that the petal-like structure found in Figure 6j–l can be ascribed to CuO/ZnO. Particularly in the FSP-P catalyst, some fluorine zeolite crystals appeared in trapezoid/plate shapes and were not entirely covered by metal oxides.

The elemental EDS mapping and image analysis of the cut surface using FIB in different scale bars, namely 1 μm to 5 μm with several ranges of magnifications from 1500× to 18,000×, were conducted for the SP, SI, CP, and FSP catalysts.

**Figure 7:**
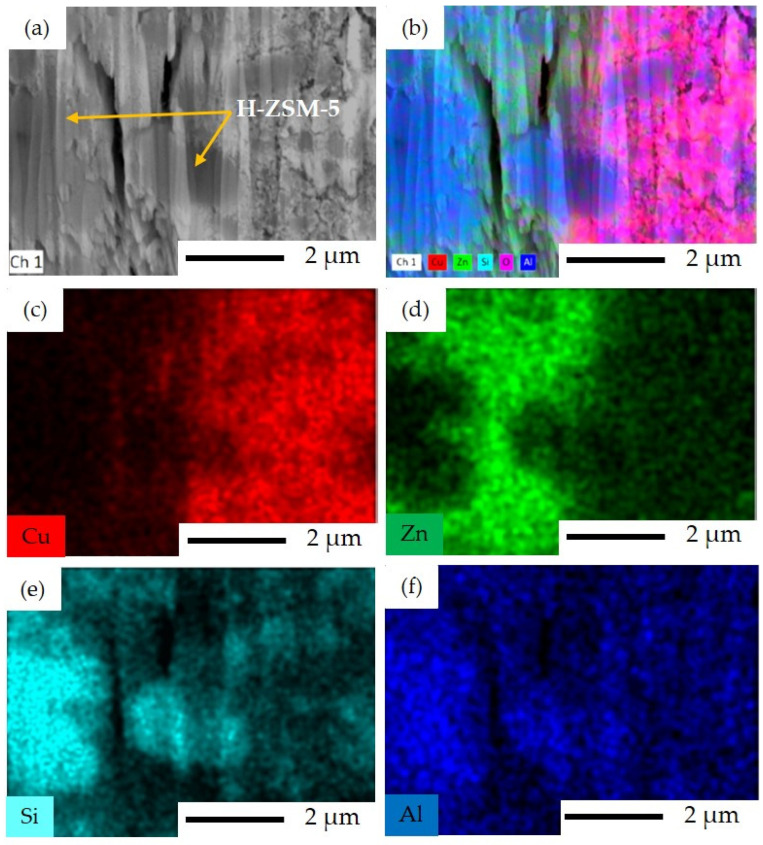
FIB-SEM image and EDS elemental mapping for SP catalyst: (**a**) FIB-SEM image of ion-cut section showing H–ZSM-5 crystals surrounded by metal oxides, (**b**) combined EDS elemental mapping of Si, Al, Cu, Zn, and O together, (**c**) EDS elemental mapping of Cu illustrating the Cu-rich area, (**d**) EDS elemental mapping of Zn illustrating the Zn-rich area, (**e**) EDS elemental mapping of Si, and (**f**) EDS elemental mapping of Al.

EDS mapping analyses of Al and Si in Figure 7e, and Figure 7f show zeolite as a component of the sequential precipitation SP catalyst. Well-dispersed Cu and Zn species across the ZSM-5 structure can also be observed here. However, two different areas with different contents of Cu and Zn can be identified, namely Cu-rich and Zn-rich areas, as seen in Figure 7c,d. 

**Figure 8:**
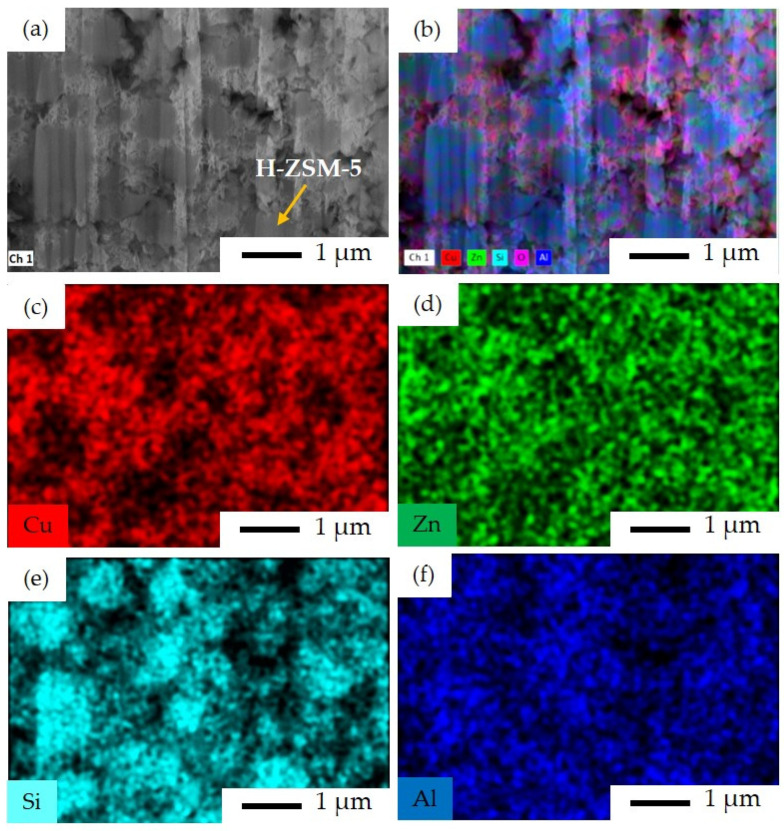
FIB-SEM image and EDS elemental mapping for SI catalyst: (**a**) FIB-SEM image of ion-cut section showing H–ZSM-5 crystals surrounded by metal oxides, (**b**) combined EDS elemental mapping of Si, Al, Cu, Zn, and O together, (**c**) EDS elemental mapping of Cu, (**d**) EDS elemental mapping of Zn, (**e**) EDS elemental mapping of Si, and (**f**) EDS elemental mapping of Al. The imaging parameters are: magnification of 18 k×, and acceleration voltage of 15 kV.

The Cu and Zn species can be seen over the surface of zeolite in the SI catalyst, as is expected from the implemented sequential impregnation method in this case (Figure 8c,d). The H–ZSM-5 crystals within the size range of 500 nm to 2 μm are covered by Cu and Zn with no visually observed agglomeration, meaning that the impregnation was successfully carried out in this catalyst. As seen in Figure 8e, the H–ZSM-5 crystals can be distinguished by their representative Si elemental content. 

**Figure 9:**
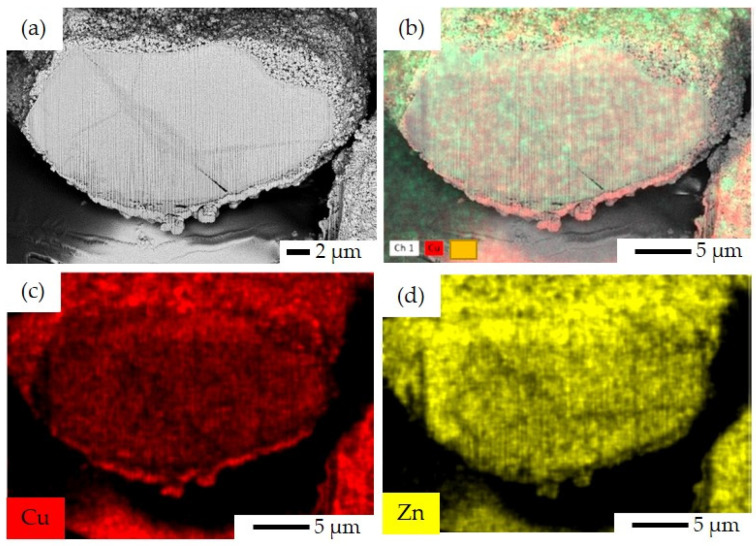
FIB-SEM image and EDS elemental mapping for CP catalyst: (**a**) FIB-SEM image of ion-cut section, (**b**) combined EDS elemental mapping of Cu and Zn together, (**c**) EDS elemental mapping of Cu, and (**d**) EDS elemental mapping of Zn.

As seen in Figure 9, the CP catalyst exhibits a large area of precipitated Cu and Zn. Although in co/sequential precipitation methods, the precipitates (metal particles) are supposed to be incorporated into the pores and channels of the zeolite structure, some metal particles might accumulate over the surface due to their bigger particle size compared to the pore size of H–ZSM-5 (Figure 7) so that the metal particles cannot diffuse into the pores easily. Despite this, the metal particles can be observed in the areas between the zeolite particles (intercrystals).

**Figure 10:**
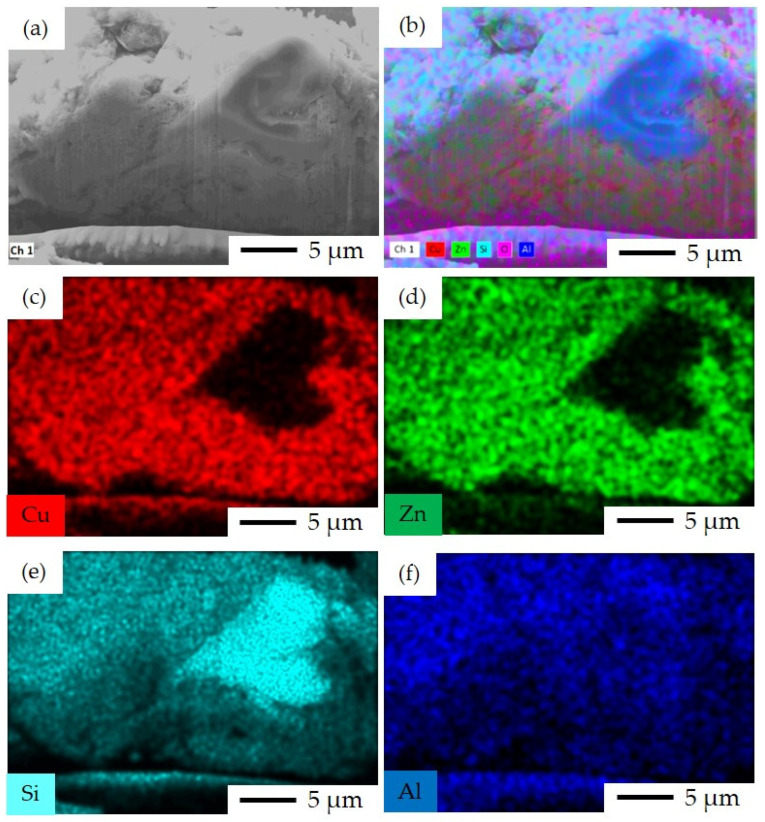
FIB-SEM image and EDS elemental mapping for FSP-P catalyst: (**a**) FIB-SEM image of ion-cut section showing fluorine ZSM-5 crystals surrounded by metal oxides, (**b**) combined EDS elemental mapping of Si, Al, Cu, Zn, and O together, (**c**) EDS elemental mapping of Cu, (**d**) EDS elemental mapping of Zn, (**e**) EDS elemental mapping of Si, and (**f**) EDS elemental mapping of Al.

FIB-SEM and EDS elemental mapping of FSP-P is shown in Figure 10. It can be observed that fluorine ZSM-5 crystals were covered by Cu and Zn oxides asymmetrically. Cu and Zn were not immigrated or diffused into the fluorine ZSM-5 crystals meaning that these crystals are free of Cu and Zn. This might be due to the synthesis limitation that the above-mentioned metal particles did not participate in the crystallization of zeolite so the crystallization solely occurred by the main relative Al and Si-based precursors. The incorporation of metal active sites in closer proximity to zeolite crystals is anticipated to enhance catalytic activity. Although fluorine ZSM-5 is formed and crystallizes in a single-pot, the limitation of controlling the formation and crystallization of metal particles hinders obtaining a closest distance between them.

**Figure 11:**
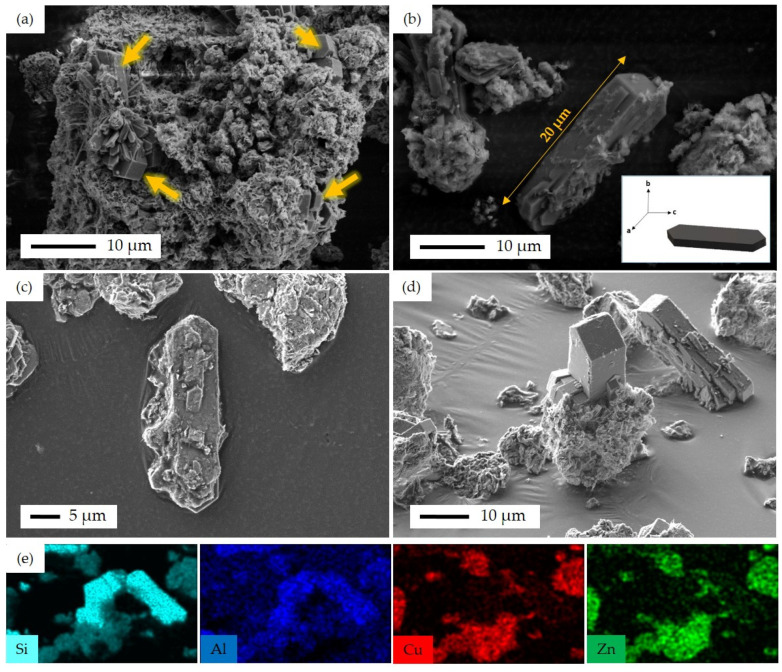
SEM images of FSP-P, (**a**) fluorine ZSM-5 crystals emerged from the metal oxide layers with different crystal sizes, as illustrated with arrows, (**b**) the largest fluorine ZSM-5 single crystal with the base of 20 μm in c-axis; insert figure shows the schematic of fluorine ZSM-5 single crystal along with the *abc*-axis, (**c**) fluorine ZSM-5 crystals without metal oxide layers, (**d**) fluorine ZSM-5 crystals emerged from the metal oxide layers, and (**e**) EDS elemental mapping of Si, Al, Cu, and Zn corresponding to (**d**).

FSP-P’s morphology was further investigated through SEM/EDS analyses. Figure 11 displays the corresponding SEM images along with elemental EDS mappings. The fluorine ZSM-5 crystals are covered with a petal-like aggregation of CuO/ZnO, as shown in Figure 11. However, some fluorine ZSM-5 crystals were not completely surrounded by CuO and ZnO and emerged from the aggregation of the metal oxides. In some areas, zeolite plates were stacked on top of one another in different directions (Figure 11a). The EDS mapping (Figure 11) reveals that the analyzed zeolite area contains only Si and Al, without Cu or Zn. This confirms a pure zeolite region, as there are no other elements extending out of the representative area. As seen, fluorine ZSM-5 crystals can be found in different sizes. For instance, in the direction of the *c*-axis, the size of zeolite crystals is between 10 μm and 20 μm, while the size varies from 1 μm to 5 μm in the direction of the *b*-axis (Figure 11a,b). Figure S9 shows the cross-section SEM images before and after cutting the FSP-P sample by Gallium ion beam in FIB-SEM. The catalytic performance of FSP might be enhanced by decreasing the distance accomplished by introducing smaller crystals, which can be established by tuning the synthesis parameters such as time, temperature, and concentration of metal particles. This requires a separate comprehensive study to be devoted primarily to analyzing this parameter. Figure S10 shows the schematic of synthesis procedures to fabricate catalysts in this work.

### 3.2. Catalytic Performance

#### 3.2.1. Effect of Reaction Temperature

The effect of varying the operating temperature (in the range of 200–260 °C) on the performance indicators of this catalytic system for two levels of operating pressure, namely 10 bar and 20 bar, are reported and discussed in this section. In comparing the catalytic performance of the catalyst with such different catalytic structural characteristics in such a wide range of operating conditions, it should be taken into account that (a) each tested set of operating conditions for some catalytic sample might be closer to the optimum set of testing conditions as for the other catalytic samples, and (b) to account for this, the observed trends, especially for each product selectivity, should be compared rather than the absolute values of performance indicators.

**Figure 12:**
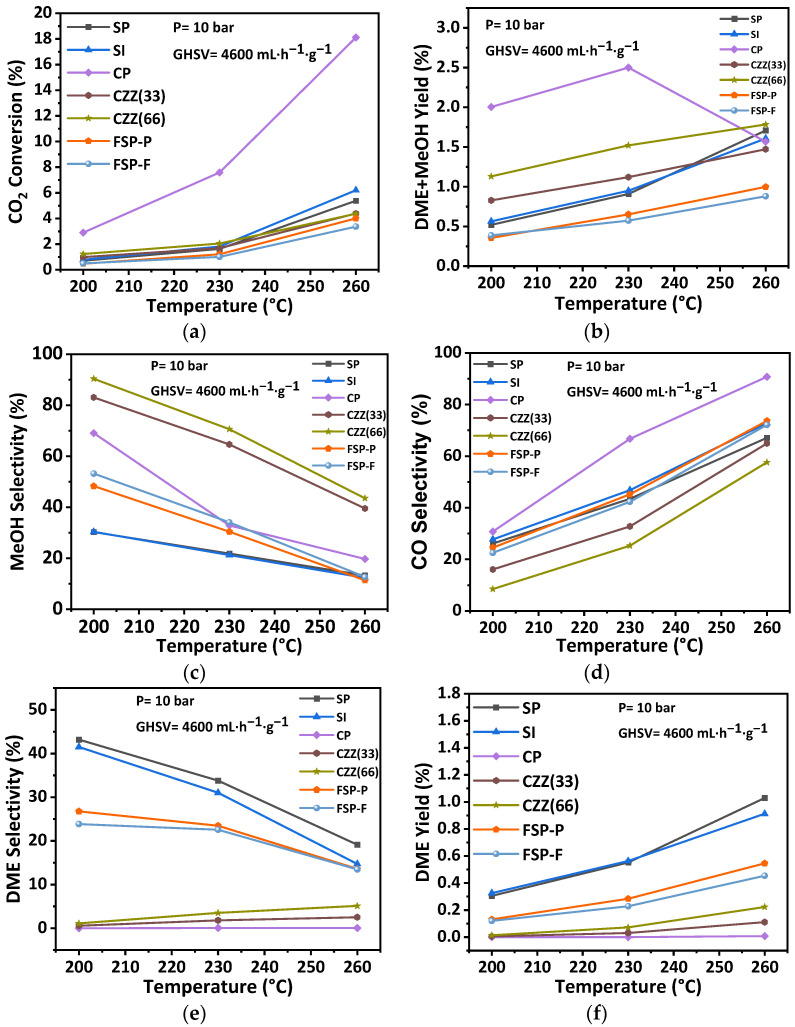
Effect of reaction temperature on catalytic performance including (**a**) CO_2_ conversion, (**b**) DME + MeOH yield, (**c**) MeOH selectivity, (**d**) CO selectivity, (**e**) DME selectivity, and (**f**) DME yield at the pressure of 10 bar, the GHSV of 4600 mL·h^−1^·g^−1^, and H_2_:CO_2_ = 3:1.

Figure 12 shows the catalytic performance at different temperatures of 200 °C, 230 °C, and 260 °C under the pressure of 10 bar, GHSV 4600 mL·h^−1^·g^−1^, and the reactants feed ratio H_2_/CO_2_ = 3. Figure 12a shows the observed trends of CO_2_ conversion for all catalysts. As expected, the rise in temperature led to an increase in CO_2_ conversion across all catalysts. It is observable that the CP catalyst showed the highest CO_2_ conversion at all temperatures with the value of about 18% at 260 °C. The highest conversion for CP can be attributed to the presence of a higher amount of accessible metal active sites in this catalyst structure (50 wt.% CuO:50 wt.% ZnO) compared to other catalysts. Apart from the CP catalyst, as CO_2_ converts over the metal active sites [49], it is expected that catalysts with a lower amount of metal active sites show a lower CO_2_ conversion. As seen in Table 4, FSP-P consists of 3.9 wt.% of CuO and 1.6 wt.% of ZnO, and CZZ(66) shows 16.7%wt of CuO and 15.9wt.% of ZnO compared to SP and SI. Further, smaller Cu crystallite sizes have been reported as providing higher CO_2_ conversion [50]. Among the other hybrid catalysts, SI and SP have the smallest Cu size with 7 nm and 8 nm, respectively, as shown in Table 4, and showed higher CO_2_ conversion compared to other catalysts. As shown in Figure 8, in the SI catalyst, Cu and Zn were distributed homogeneously across the zeolite crystals, which can be another reason why CO_2_ conversion for this catalyst is higher than the other ones. In addition, in FSP-P and CZZ(66) catalysts, the amount of metal active sites is lesser than active sites found in CP catalysts, so the access to the metal active sites in these catalysts is more restricted than those in CP catalysts. However, CZZ(33) and CZZ(66) catalysts showed a similar trend even though in the CZZ(33) catalyst, a higher amount of metal active sites is present (with 35.8%wt of CuO and 31.5%wt ZnO) compared to the CZZ(66) catalyst. Although the SI catalyst with 11%wt CuO possesses a lower amount of metal contents than the SP catalyst with 20%wt CuO, it showed slightly higher CO_2_ conversion, especially at the highest temperature (i.e., 260 °C). The reason might be attributed to the accessibility of CuO active sites considering that a sequential precipitation method was applied in the SP catalyst to introduce metal contents into the zeolite structure. In this manner, the ZnO precursor was initially precipitated inside the pores, then the CuO precursor was introduced to precipitate into the zeolite structure. In contrast, the sequential impregnation method was applied for the SI catalyst to place the metal active sites on the zeolite surface. That way, ZnO particles were impregnated on zeolite, followed by CuO’s impregnation, establishing a second layer on the previous ZnO layer. In this case, the CuO active sites have better access to the reactants than the active sites in the SP catalyst. As seen in Figure 12c, by increasing the temperature, the DME selectivity has decreased for some of the catalysts, including SP, SI, and FSP catalysts. At the same time, it has been almost constant for CZZ(33), CZZ(66), and CP catalysts. The explanation for the observed reduction in the DME selectivity can be provided with a focus on the exothermic behavior of methanol dehydrogenation that leads to the decline in DME selectivity by increasing the temperature [21]. As reported elsewhere, methanol dehydration takes place over the acid sites with weak and medium strength in a manner that the weak acid sites increase the mobility of protons in the zeolites [51]. Therefore, the existence of these acid sites in the solids can be attributed to higher DME selectivity. In a study conducted by Poreddy et al. [52], it was found that zeolites impregnated by CuO show two extra NH_3_ desorption peaks that can increase the medium acid sites. It has also been reported that acid peaks in the NH_3_–TPD profile are influenced by copper loadings higher than 2.5 wt.% increasing strong acid sites [52]. As seen in Figure 12, SP and SI catalysts showed higher DME selectivity, which is in accordance with the NH_3_–TPD results (Figure S7) that show larger weak and medium acid areas compared to the other catalysts. Unlike these catalysts, CZZ(33) and CZZ(66) showed lower weak and medium acid sites even lower than the FSP-P catalyst, which exhibited higher weak acid compared to CZZ(33) and CZZ(66) so that the DME selectivity of FSP catalysts is higher. Meanwhile, CO selectivity has significantly increased by increasing the temperature for all catalysts. This can be attributed to the impact of the endothermic RWGS reaction [21]. Increasing the temperature increased the DME yield. SP and SI catalysts showed higher DME yield than the FSP-P, FSP-F, and CP catalysts since the zeolite content is higher for these catalysts, as shown in Table 4. For the CP catalyst, the DME yield is zero due to the absence of zeolites in this catalyst. It was also observed that not all of the zeolite crystals emerge in FSP-P, and FSP-F catalysts are surrounded by Cu and Zn species. This is another reason why higher DME yields have been observed for those catalysts. Furthermore, since zeolite is responsible for methanol dehydration in the DME [28], emerging zeolites on the surface of the catalyst can lead to an increase in DME yield. Figure S11 compares the DME yield at 260 °C with an intensity of Si + Al, as zeolite representatives, extracted from XPS data in Figure 3. As seen, DME yield increases by increasing the amount of Si + Al near the surface of the catalyst. In addition, DME yield data are in accordance with the BET results presented in Table 5 in which SP and SI showed a higher surface area compared to the other catalysts following that they showed a higher DME yield.

For the single-pot synthesized catalyst FSP, since the closest distance between the metal active site and zeolite acid sites is expected, a better interaction between the hydrogenation and methanol dehydration reactions could be established. However, the experienced restrictions in tuning the synthesis conditions and introducing the metal active sites during the single-pot procedure do not allow for establishing the catalyst composition with the proper size of zeolite crystals and high portions of metal contents. Considering this, only 16% of each metal oxide (CuO and ZnO) could successfully be introduced in the FSP catalyst while simultaneously synthesizing the zeolite metal oxide structure. Figure S2 shows the XRD pattern of the catalyst obtained through one of the unsuccessful applied single-pot syntheses. Table S3 represents the data obtained for the catalytic performance testing of all catalysts at different temperatures of 200 °C, 230 °C, and 260 °C under the pressure of 10 bar, GHSV of 4600 mL·h^−1^·g^−1^, and H_2_/CO_2_ = 3. 

The general observation was that the CP catalyst containing solely CuO–ZnO, which has been tailored for CO_2_ hydrogenation to methanol [28], provides the highest conversion of CO_2_ as the distribution of active materials there, as well as its chemical–structural characteristics, secure the highest CO_2_ conversion. Nevertheless, this does not necessarily come with the highest selectivity towards methanol (indicated by the recorded highest CO-selective conversion in Figure 12d) and indeed not towards DME. Higher selectivity towards DME can be secured by introducing ZSM-5 into the hybrid catalytic structure if this is carried out adequately on the nanometer–micrometer scale. This is achieved using different combinations of precipitation and impregnation synthesis, where SP, SI, and even FSP catalysts have shown the best DME yield (Figure 12f). An attempt was made to secure a smaller distance using single-pot synthesis in the micrometer scale. In fact, crafting the active materials (CuO–ZnO and ZSM-5) beyond this scale, as has been established using the electrospun of these separate materials (CZZ(33) and CZZ(66)) in fiber forms, does not provide the right dynamic between the catalytic hydrogenation and methanol dehydration. As seen in Figure 12e, a very low level of DME yield has been obtained using those catalytic samples. By taking into account calcination temperatures for all samples, it is expected that the FSP-P and FSP-F show the lowest DME selectivity, while the CZZ(33) and CZZ(66) with a calcination temperature of 550 °C are lower than the FSP catalysts with higher calcination temperatures. Considering the observed trends in Figure 12d, it can be concluded that the impact of operating temperature on the CO selectivity of all catalyst samples is significant. Moreover, the catalyst showing the highest CO_2_ conversion (e.g., CP catalyst) usually results in the highest CO selectivity, as this system’s trade-off between the conversion and desired selectivity is known. Introducing zeolites and increasing the zeolite content of the catalyst reduces both the CO_2_ conversion and the CO selectivity, as seen, for instance, by comparing the trends in Figure 12d for the CZ33 and CZ66 catalyst samples.

**Figure 13:**
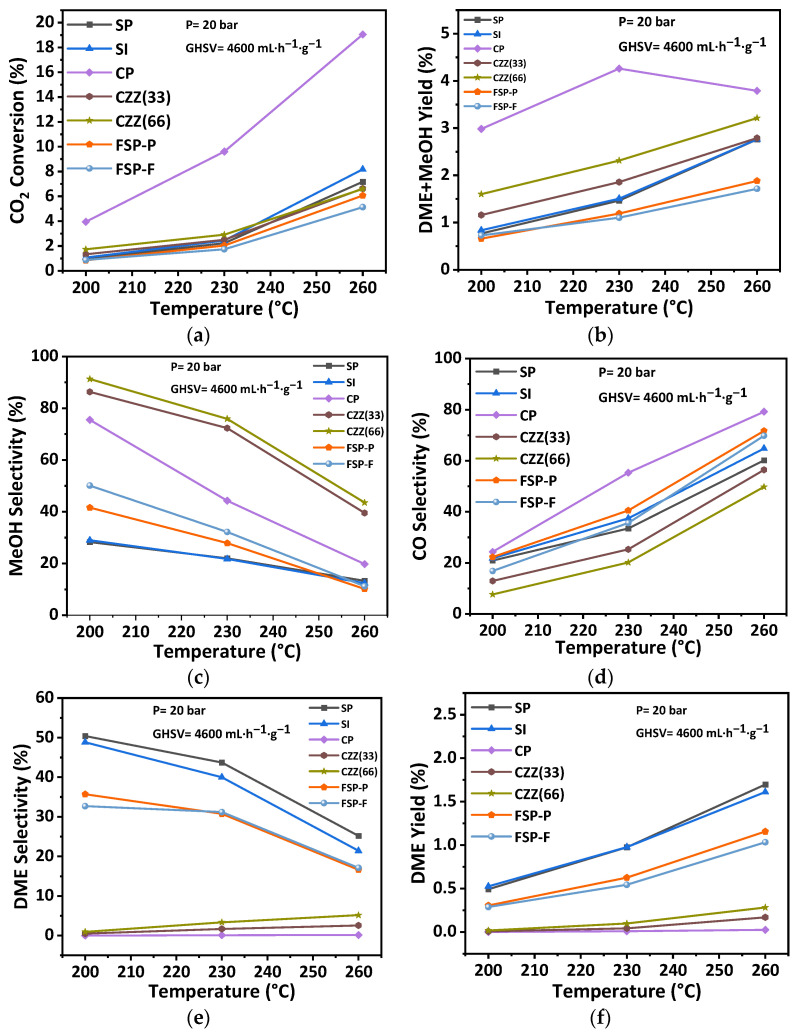
Effect of reaction temperature on catalytic performance including (**a**) CO_2_ conversion, (**b**) DME + MeOH yield, (**c**) MeOH selectivity, (**d**) CO selectivity, (**e**) DME selectivity, and (**f**) DME yield at the pressure of 20 bar, the GHSV of 4600 mL·h^−1^·g^−1^, and H_2_:CO_2_ = 3:1.

Similarly, the recorded trends and the values of the catalytic performance indicators under 20 bar pressure while varying the operating temperature are reported in this section and the rest in Table S4. This enables the investigation of the effect of reaction temperature (200 °C, 230 °C, and 260 °C) at two different pressures, with the aim of reflecting an interactive effect of the temperature and pressure on the catalytic performance. Therefore, all catalysts were also tested at the pressure of 20 bar, H_2_:CO_2_ = 3:1, and GHSV of 4600 mL·h^−1^·g^−1^. Figure 13 presents the correspondingly recorded catalytic performance indicators. As seen in Section 3.2 (P = 10 bar, GHSV = 4600 mL·h^−1^·g^−1^), a similar trend can be observed for the recorded catalytic performance at 20 bar. As seen in Figure 13, CO_2_ conversion was increased to a slightly higher value by increasing the temperature. Meanwhile, the rate of increase in CO selectivity was reduced at a higher pressure (i.e., 20 bar). It indicates the enhancing effect of pressure in decreasing CO selectivity in such catalytic systems. The trend of methanol selectivity against temperature in Figure 13c, at the higher pressure, was almost constant compared to the trend obtained at a lower pressure of 10 bar (Figure 12c). The DME selectivity is constant for the CP catalyst at all temperatures, while it slightly increased for CZZ(33) and CZZ(66) (Figure 13e). The observed increase in DME selectivity for the CZZ(66) is, as expected: higher than for the CZZ(33) due to its higher zeolite content. DME selectivity in the SP, SI, FSP-P, and FSP-F catalysts is high, following a similar trend as methanol selectivity, and reduces while the temperature increases. The highest DME yield has been achieved through these structures, implying that the reactants are equally accessible to the zeolite material as they are to the CuO–ZnO active sites. This is valuable information for tailoring the composition and selecting the synthesis method for such a hybrid catalytic system. The results in this section show that the catalytic performance of CO_2_ hydrogenation to DME can be improved at higher pressures, as the reaction of CO_2_ hydrogenation to methanol is favorable at high pressures by decreasing the number of moles [53,54]. Similar to the trend obtained in the previous section, the DME yield increased after increasing the temperature at a higher pressure of 20 bar. 

#### 3.2.2. Effect of GHSV

**Figure 14:**
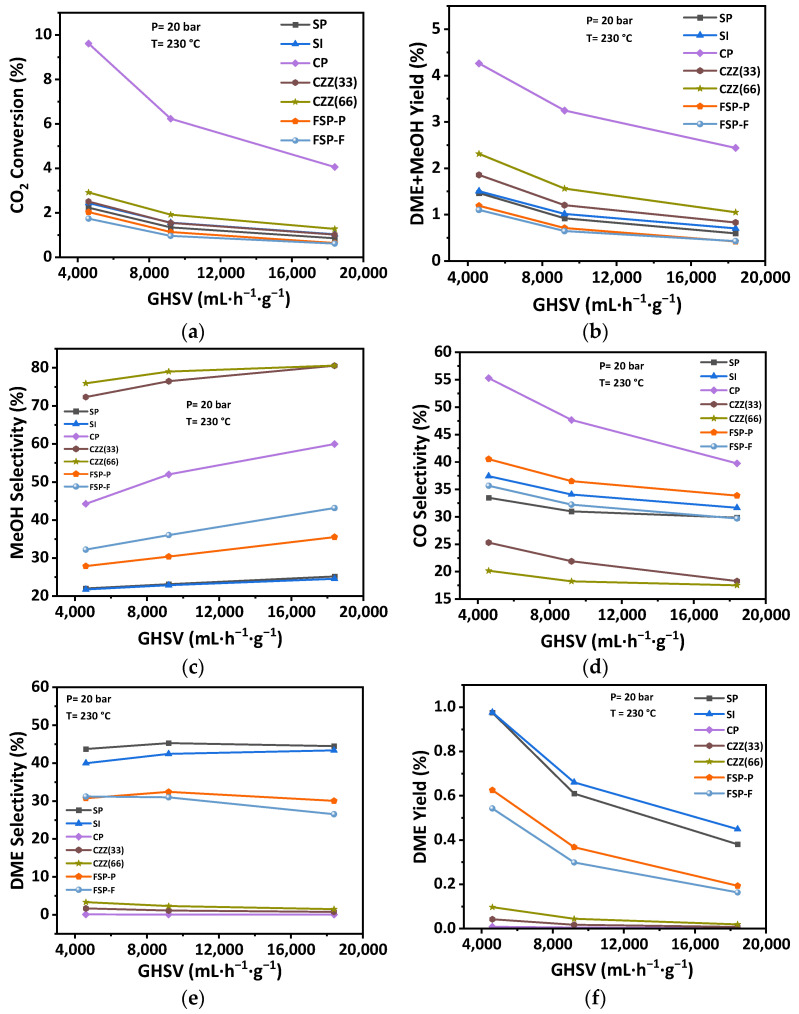
Effect of GHSV on the catalytic performance including (**a**) CO_2_ conversion, (**b**) DME + MeOH yield, (**c**) MeOH selectivity, (**d**) CO selectivity, (**e**) DME selectivity, and (**f**) DME yield at the pressure of 20 bar, the temperature of 230 °C, and H_2_:CO_2_ = 3:1.

Observing that the highest operating pressure enhances the catalytic performance in terms of selectivity and CO_2_ conversion, the effect of GHSV was studied at 20 bar. The catalytic performance was also carried out at three different GHSVs of 4600 mL·h^−1^·g^−1^, 9200 mL·h^−1^·g^−1^, and 18,400 mL·h^−1^·g^−1^. Figure 14 represents the catalytic performance of catalysts as a function of GHSV at P = 20 bar and for the average temperature T = 230 °C, and stoichiometric ratio H_2_:CO_2_= 3. CO_2_ conversion was decreased by increasing the GHSV. The declining rate for all catalysts was lower at higher GHSVs of 9200 mL·h^−1^·g^−1^ and 18,400 mL·h^−1^·g^−1^; the conversion remained constant by varying the GHSV. As can be seen in Figure 14e, DME selectivity is slightly increased for the SP and SI catalysts at 4600 mL·h^−1^·g^−1^, but becomes constant by increasing the GHSV. The DME selectivity for CZZ(33), CZZ(66), and CP is slightly decreased at higher GHSVs. Methanol selectivity of all catalysts was increased by increasing the GHSV. In contrast, CO selectivity is reduced by increasing the GHSV. 

The variation in GHSV from 9200 mL·h^−1^·g^−1^ to 18,400 mL·h^−1^·g^−1^ does not significantly change the catalytic performance, though. The DME yield declined by increasing the GHSV. Again, in this part of sensitivity analysis, SP and SI catalysts exhibit a higher DME yield compared to other catalysts. FSP-P and FSP-F catalysts also show a relatively high DME yield. In fact, both the DME yield and DME + methanol yield decrease by increasing GHSV, indicating again that accessibility of the reactants to CuO–ZnO catalytic materials is key. Reviewing the reported surface areas associated with the CuO–ZnO material (Table 4) and the effect of increasing GHSV on reducing the DME + methanol yield also confirms this. Here, also as expected, the catalyst showing the highest CO_2_ conversion (e.g., CP catalyst) usually results in the highest CO selectivity. Introducing zeolites and increasing the zeolite content of the catalyst reduce both the CO_2_ conversion as well as the CO selectivity, as seen, for instance, by comparing the trends in Figure 14d for the CZ33 and CZ66 catalyst samples. Therefore, using a single-pot synthesis and electrospinning the resulting hybrid material, especially when those procedures are optimized, could address the challenge of introducing zeolite material into the hybrid catalyst and minimizing its undesired effect in reducing the reactants’ accessibility to the hydrogenation catalytic materials (i.e., CuO–ZnO in this research). All trends based on the catalytic performance indicators at different GHSVs are reported in Table S5. The reproducibility data for repeated catalysts are shown in Figures S12–S14. Moreover, Arrhenius plots for all catalysts can be found in Figure S15.

## 4. Conclusions

In this paper, the methodology and the results of a systematic analysis are presented as a step towards ultimately correlating the chemical–structural characteristics and performance of the hybrid catalytic materials for CO_2_ hydrogenation to DME. This was accomplished via synthesizing, characterizing, and testing the performance of several catalytic structures. In this manner, the challenges associated with establishing a selective-efficient hybrid catalytic structure composed of CuO–ZnO and ZSM-5, respectively, responsible for CO_2_ hydrogenation to methanol and methanol dehydration to DME, were identified and analyzed. In particular, the accessibility of reactants to the catalytic sites could be restricted by introducing the zeolite material with a micropore structure, primarily due to water condensation in such small pores. Utilizing a selected set of operating pressure, temperature, and contact time, as well as establishing the material–structural characteristics to secure a significant methanol production to be quickly dehydrated to DME, was the ultimate goal and the guide in using different combinations of precipitation and impregnation of the hydrogenation and dehydration catalytic materials with and without being electrospun. It was demonstrated that co-precipitation of all involved precursors to form CuO, ZnO, and ZSM-5 in a single-pot synthesis of such hybrid catalyst could be a part of the ultimately selected synthesis strategy. The size of the zeolite crystals on the micro-scale of such a hybrid catalyst should be optimized by fine-tuning such a synthesis procedure so that a fine distribution and close vicinity of the copper and zinc species and the zeolite crystals could be secured. Moreover, using the electrospinning technique enables improving the reactants’ access to the active materials in a fiber-form catalytic structure, thereby enhancing selective DME production. These were observed and analyzed using the results of a comprehensive catalytic characterization and testing study. The observed behaviors and the involved phenomena in the scale of catalyst particles and fibers (in the range below 1 micrometer), and testing their cluster (in the scale of tens of microns) or the whole catalytic bed (in mm scale), were analyzed and explained to be possibly used in guiding the future efforts in tailoring an efficient catalytic structure for this system. Moreover, the Arrhenius plots reflecting the activity of the catalysts along with the recorded trends of CO_2_ conversion and product selectivity along the time reflecting their stability were presented. The fresh and spent catalysts were further analyzed and their characteristics were compared using XPS and SEM analyses. No significant change was observed in the morphology of spent catalysts in comparison to the fresh catalysts.

## Figures and Tables

**Table 1:** Chemicals used in the catalysts’ synthesis.

Chemicals	Chemical Formula	Molecular Weight (g·mol^−1^)	Purity (%)	Supplier
PVP (Polyvinylpyrrolidone)	(C_6_H_9_NO)_n_	1,300,000	N/A ^1^	Sigma-Aldrich, Saint Louis, MO, USA
PVP (Polyvinylpyrrolidone)	(C_6_H_9_NO)_n_	40,000	N/A	Sigma-Aldrich, Saint Louis, MO, USA
DMF (N,N-Dimethylformamide)	HCON(CH_3_)_2_	73.09	99.5	Sigma-Aldrich, Saint Louis, MO, USA
Copper(II) nitrate trihydrate	Cu(NO_3_)_2_x3H_2_O	241.60	≥99.5	Merck, Darmstadt, Germany
Zinc nitrate hexahydrate	Zn(NO_3_)_2_x6H_2_O	297.49	≥99.0	Honeywell, Muskegon, MI, USA
H–ZSM-5 (SiO_2_/Al_2_O_3_ = 30/1)	(SiO_2_)_30_(Al_2_O_3_)H^+^	N/A (3.4 μm av. Particle)	N/A	Alfa-Aesar, Kandel, Germany
Fumed silica	SiO_2_	60.08	≥99.0	AEROPERL, Hanau, Germany
Aluminum nitrate nonahydrate	Al(NO_3_)_3_x9H_2_O	375.13	98.5	Merck, Darmstadt, Germany
TPAOH (Tetra Propyl Ammonium Hydroxide)	(CH_3_CH_2_CH_2_)_4_N(OH) (1 Molar in H_2_O)	203.36	N/A	Sigma-Aldrich, Saint Louis, MO, USA
Ammonium fluoride	NH_4_F	37.04	95	Merck, Darmstadt, Germany

^1^ Not Available.

**Table 2:** The list, codes, and specifications of the synthesized tested catalysts in this research ^1^.

Catalyst Code	Material System	Desired Structure	Composition (wt.%)	Preparation Method	Calcination Temperature (°C)
CP	CuO–ZnO	Powder	50%CuO:50%ZnO	Co-precipitation	360
SP	H–ZSM-5/CuO–ZnO	Powder	33.3%CuO:33.3%ZnO:33.3%H–ZSM-5	Sequential precipitation	360
SI	H–ZSM-5/CuO–ZnO	Powder	16.7%CuO:16.7%ZnO:66.6%H–ZSM-5	Sequential impregnation	360
H–ZSM-5-P ^2^	H–ZSM-5	Powder	100%H–ZSM-5-P	Commercial	N/A
H–ZSM-5-F ^3^	H–ZSM-5	Fiber	100%H–ZSM-5-P	Electrospinning	550
CZZ(33%)	H–ZSM-5/CuO–ZnO	Fiber	33.3%CuO:33.3%ZnO:33.3%H–ZSM-5	Electrospinning	550
CZZ(66%)	H–ZSM-5/CuO–ZnO	Fiber	16.7%CuO:16.7%ZnO:66.6%H–ZSM-5	Electrospinning	550
FSP-P	Fluorine-ZSM-5/CuO–ZnO	Powder	16.7%CuO:16.7%ZnO:66.6%H–ZSM-5	Single-pot	800
FSP-F	Fluorine-ZSM-5/CuO–ZnO	Fiber	16.7%CuO:16.7%ZnO:66.6%H–ZSM-5	Electrospinning	550

^1^ All tested catalysts were diluted using 2250 mg of Silicon Carbide (SiC) in the packed-bed. ^2^ Commercial powder of H–ZSM-5. ^3^ This was used only as a reference for comparing the material and structural characteristics.

**Table 3:** DOE of the catalytic activity test.

Step	GHSV	Gas Flow Each Reactor (H_2_:CO_2_:N_2_) Total Flow	Temperature	Pressure (abs)
Units	Approx. mL·h^−1^·g^−1^	NmL·min^−1^	°C	Bar
0	-	Reduction step (50%H_2_:50%N_2_) 40	300	1
0	-	Cooling under N_2_ 40	260	1
1	4600	(64%H_2_:21%CO_2_:15%N_2_) 25	260	20
2	4600	(64%H_2_:21%CO_2_:15%N_2_) 25	260	10
3, repro 1	4600	(64%H_2_:21%CO_2_:15%N_2_) 25	260	20
4	4600	(64%H_2_:21%CO_2_:15%N_2_) 25	230	20
5	4600	(64%H_2_:21%CO_2_:15%N_2_) 25	230	10
6	9200	(64%H_2_:21%CO_2_:15%N_2_) 50	230	20
7	18,400	(64%H_2_:21%CO_2_:15%N_2_) 100	230	20
8	4600	(64%H_2_:21%CO_2_:15%N_2_) 25	200	10
9	4600	(64%H_2_:21%CO_2_:15%N_2_) 25	200	20

**Table 4:** The phase composition and average crystallite size of ZSM-5 and CuO for powder and fiber samples as determined by XRD Rietveld refinement, ICP.

Sample	Composition (wt.%) ^1^	XRD Phase Composition (wt.%) ^2^			Crystallite Size (nm) ^2^		Cu/Zn ^3^
		ZSM-5	CuO	ZnO	ZSM-5	CuO	
H–ZSM-5-P	100%H–ZSM-5-P	100	0	0	57	-	0
SP	33.3%CuO:33.3%ZnO:33.3%H–ZSM-5	80.6	11	5.5	55	8	1.17
SI	16.7%CuO:16.7%ZnO:66.6%H–ZSM-5	74.7	20	5.2	57	7	1.01
CZZ(33)	33.3%CuO:33.3%ZnO:33.3%H–ZSM-5	32.8	35.8	31.5	40	28	1.12
CZZ(66)	16.7%CuO:16.7%ZnO:66.6%H–ZSM-5	67.4	16.7	15.9	36	12	1.04
FSP-P	16.7%CuO:16.7%ZnO:66.6%H–ZSM-5	63.4	3.9	1.6	44	22	1.5

^1^ Intended composition in wt.% ^2^ Calculated by Rietveld refinement. ^3^ Derived from ICP analysis.

**Table 5:** Textural properties of all samples.

Catalyst/Sample	Composition (wt.%)	S_BET_ (m^2^·g^−1^) ^1^	S_external_ (m^2^·g^−1^) ^2^	S_micropore_ (m^2^·g^−1^) ^3^	V_micropore_ (cm^3^·g^−1^) ^4^	V_mesopore_ (cm^3^·g^−1^) ^4^	Pore Size (nm) ^5^
CP	50% CuO:50% ZnO	40.9	38.1	2.8	0.001	0.11	3.78
SP	33.3% CuO:33.3%ZnO:33.3% H–ZSM-5	183	41.1	141.9	0.07	0.10	1.76
SI	16.7% CuO:16.7% ZnO:66.6% H–ZSM-5	191.7	42.6	149.2	0.073	0.18	1.54
CZZ(33)	33.3% CuO:33.3% ZnO:33.3% H–ZSM-5	26.1	25.3	0.8	0.001	0.12	1.61
CZZ(66)	16.7% CuO:16.7% ZnO:66.6% H–ZSM-5	132	35.8	96.1	0.048	0.18	1.68
FSP-P	16.7% CuO:16.7% ZnO:66.6% H–ZSM-5	91.8	42.04	49.8	0.025	0.25	1.68
FSP-F	16.7% CuO:16.7% ZnO:66.6% H–ZSM-5	69. 6	51.1	18.5	0.01	0.19	1.61
H–ZSM-5-P	16.7% CuO:16.7% ZnO:66.6% H–ZSM-5	308.1	51.04	257.04	0.13	0.14	1.76
H–ZSM-5-F	16.7% CuO:16.7% ZnO:66.6% H–ZSM-5	324.6	71.5	253.1	0.12	0.15	1.76

^1^ BET surface area. ^2^ External surface area derived from the t-plot method. ^3^ Microporous volume and surface area were both calculated from the t-plot. ^4^ Mesoporous volume calculated by subtracting microporous volume from the total volume. ^5^ Pore diameter was calculated using BJH method.

## Data Availability

All data supporting the findings of this study are available in this article and the Supplementary Materials.

## Supplementary Materials

The following supporting information can be downloaded at https://www.mdpi.com/article/10.3390/ma16237255/s1, Figure S1: Picture of ES device used in this study to produce fiber catalysts vs the schematic of the device; Figure S2: XRD patterns of SP-1 and acidic-ZSM-5 powders, SP-1 in the present of metal nitrates; Figure S3: TGA curve corresponding to FSP-P; Figure S4: waterfall XRD pattern of FSP calcined at different temperatures of 500 °C, 600 °C, 650 °C, and 800 °C. H–ZSM-5-Com is used as a reference sample; Figure S5: XPS of C 1 s for all samples; Figure S6: XPS spectra of Cu 2p for fresh and spent catalysts of (a) CP and CP-T, (b) SI and SI-T, and (c) FSP-P and FSP-P-T; Figure S7: NH_3_–TPD profile for all catalysts and H–ZSM-5-P for comparison; Table S1: the Cu 2p XPS feature peaks, corresponding chemical shifts, and Cu^2+^ concentration; Table S2: the Zn 2p XPS feature peaks and corresponding intensities; Figure S8: EDS analysis of CZZ(33) fibers after calcination; Figure S9: (a) SEM image of FSP-P; the cut section shown, (b) FIB-SEM of FSP-P after cutting by Gallium ion beam (EDS elemental mapping of this image is shown in Figure 11); Figure S10: synthesis and preparation procedures schematic of (a) CP reference powder catalyst, (b) SP powder catalyst, (c) SI powder catalyst, (d) CZZ(33) and CZZ(66) fibrous catalysts, and (e) FSP-P powder catalyst and (f) FSP-F fibrous catalyst; Figure S11: DME yield at 260 °C and XPS intensity of Si + Al as zeolite representatives; Figure S12: reproducibility results for effect of reaction temperature on catalytic performance including (a) CO_2_ conversion, (b) DME + MeOH yield, (c) MeOH selectivity, (d) CO selectivity, (e) DME selectivity, and (f) DME yield at the pressure of 10 bar, the GHSV of 4600 mL·h^−1^·g^−1^, and H_2_:CO_2_ = 3:1; Figure S13: reproducibility results for the effect of reaction temperature on catalytic performance including (a) CO_2_ conversion, (b) DME + MeOH yield, (c) MeOH selectivity, (d) CO selectivity, (e) DME selectivity, and (f) DME yield at the pressure of 20 bar, the GHSV of 4600 mL·h^−1^·g^−1^, and H_2_:CO_2_ = 3:1; Figure S14: reproducibility results for the effect of GHSV on the catalytic performance including (a) CO_2_ conversion, (b) DME + MeOH tield, (c) MeOH selectivity, (d) CO selectivity, and (e) DME selectivity at the pressure of 20 bar, the temperature of 230 °C, and H_2_:CO_2_ = 3:1; Table S3: catalytic performance for all catalysts at different temperatures under 10 bar, the GHSV of 4600 mL·h^−1^·g^−1^, and H_2_:CO_2_ = 3:1; Table S4: catalytic performance for all catalysts at different temperatures under 20 bar, the GHSV of 4600 mL·h^−1^·g^−1^, and H_2_:CO_2_ = 3:1; Table S5: catalytic performance for all catalysts at different GHSVs under 20 bar, the temperature of 230 °C, and H_2_:CO_2_ = 3:1; Figure S15: Arrhenius plots of all powder and fibrous catalysts including CP, SP, SI, CZZ(33), CZZ(66), FSP-P, and FSP-F (a) at the pressure of 10 bar and the GHSV of 4600 mL·h^−1^·g^−1^, and (b) at the pressure of 20 bar and the GHSV of 4600 mL·h^−1^·g^−1^; Figure S16: stability of the catalysts as time on stream plots for (a) CO_2_ conversion, (b) DME selectivity, and (c) MeOH selectivity; Figure S17: SEM images of fresh and spent catalysts of (a) SP, (b) SP-T, (c) SI, (d) SI-T, (e) FSP-P, and (f) FSP-P-T; references [52,55,56,57,58,59,60,61] are cited in the Supplementary Materials.

## Appendix A

**Table A1:** All used abbreviations in this work along with the corresponding expression.

Abbreviation	Expression
BE	Binding Energy (the energy required to remove an electron from the sample)
BET	Brunauer–Emmett–Teller (a model for calculating the specific surface area)
BJH	Barrett–Joyner–Halenda (a model for obtaining pore-size distribution)
CP	Co-Precipitated Catalyst
CZZ	CuO–ZnO/ZSM-5 Electrospun Fibers
DI	De-Ionized (water)
DME	DiMethyl Ether (Methoxymethane)
DMF	N,N-Dimethylformamide
DOE	Design Of Experiment
EDS	Energy-Dispersive X-ray Spectroscopy
ES	Electrospinning
F	Fiber
FIB	Focused Ion Beam
FSP	Fluorine-assisted Single-Pot CuO–ZnO/ZSM-5
GC	Gas Chromatography
ICP-AES	Inductively Coupled Plasma Atomic Emission Spectroscopy
MeOH	Methanol (CH_3_OH)
P	Powder
PVP	Polyvinylpyrrolidone
SEM	Scanning Electron Microscope
SI	Sequential Impregnated Catalyst
SP	Sequential Precipitated Catalyst
TPAOH	Tetra Propyl Ammonium Hydroxide
TPD	Temperature Programmed Desorption
XRD	X-Ray Diffraction
XPS	X-ray photoelectron spectroscopy

**Table A2:** Symbols used in this work with the description.

Symbols	Description	Unit
GHSV	Gas Hourly Space Velocity (under standard conditions)	$h^{-1}$
Mw	Molecular weight	$g\cdot {mol}^{-1}$
P	Pressure	bar
Q	Total flow rate	$NmL\cdot {min}^{-1}$
S	Selectivity	%
T	Temperature	°C
X	CO_2_ conversion	%
x	Mole fraction	-
Y	Yield	%
