# Supplementary material for: Hydrogenation of Carbon Dioxide to Dimethyl Ether on CuO–ZnO/ZSM-5 Catalysts: Comparison of Powder and Electrospun Structures
Source: Materials (Basel). 2023 Nov 21;16(23):7255. doi: 10.3390/ma16237255 (PMC10707656; doi:10.3390/ma16237255)
Supplement: Supplementary file 1 [file materials-16-07255-s001.zip › materials-2651765-supplementary.pdf]

Supplementary information for

Hydrogenation of Carbon Dioxide to Dimethyl Ether on CuO–ZnO/ZSM-5 Catalysts: Comparison of Powder and Electrospun Structures

Aidin Nejadsalim [1], Hamid Reza Godini [2,*], Sanjay Ramesh Kumar [2], Fausto Gallucci [2], Delf Kober [1], Aleksander Gurlo [1] and Oliver Görke [1,*]

1 Technische Universität Berlin, Faculty III—Process Sciences, Institute of Material Science and Technology, Chair of Advanced Ceramic Materials, Straße des 17. Juni 135, 10623 Berlin, Germany; aidin.nejadsalim@ceramics.tu-berlin.de (A.N.); delf.kober@ceramics.tu-berlin.de (D.K.); gurlo@ceramics.tu-berlin.de (A.G.)

2 Inorganic Membranes and Membrane Reactors, Sustainable Process Engineering, Chemical Engineering and Chemistry, Eindhoven University of Technology, 5612 AZ Eindhoven, The Netherlands; sanjayramesh014@gmail.com (S.R.K.); f.gallucci@tue.nl (F.G.)

* Correspondence: hamid@carbon.one (H.R.G.); oliver.goerke@ceramics.tu-berlin.de (O.G.)

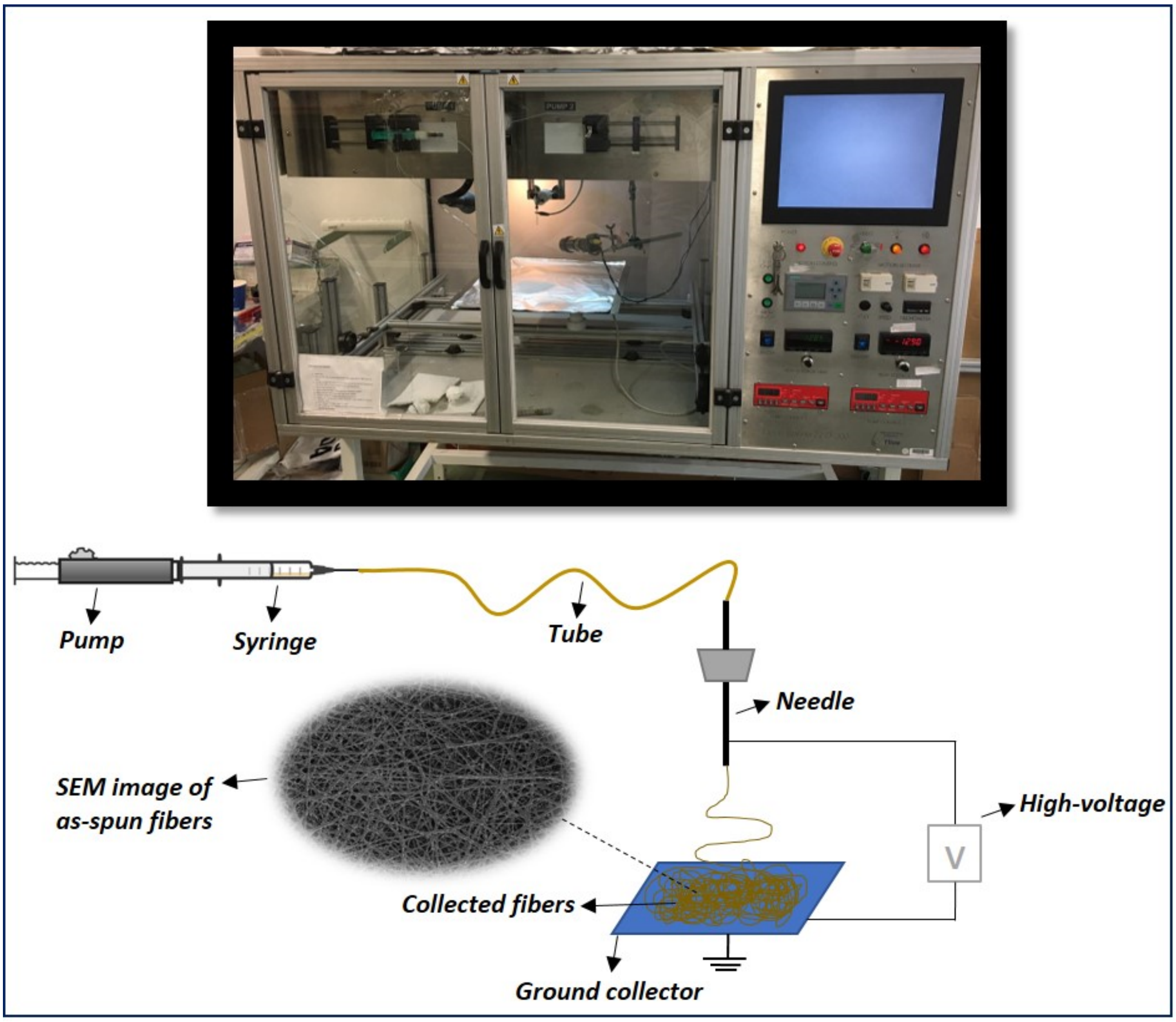

Figure S1. Picture of ES device used in this study to produce fiber catalysts vs the schematic of the device.

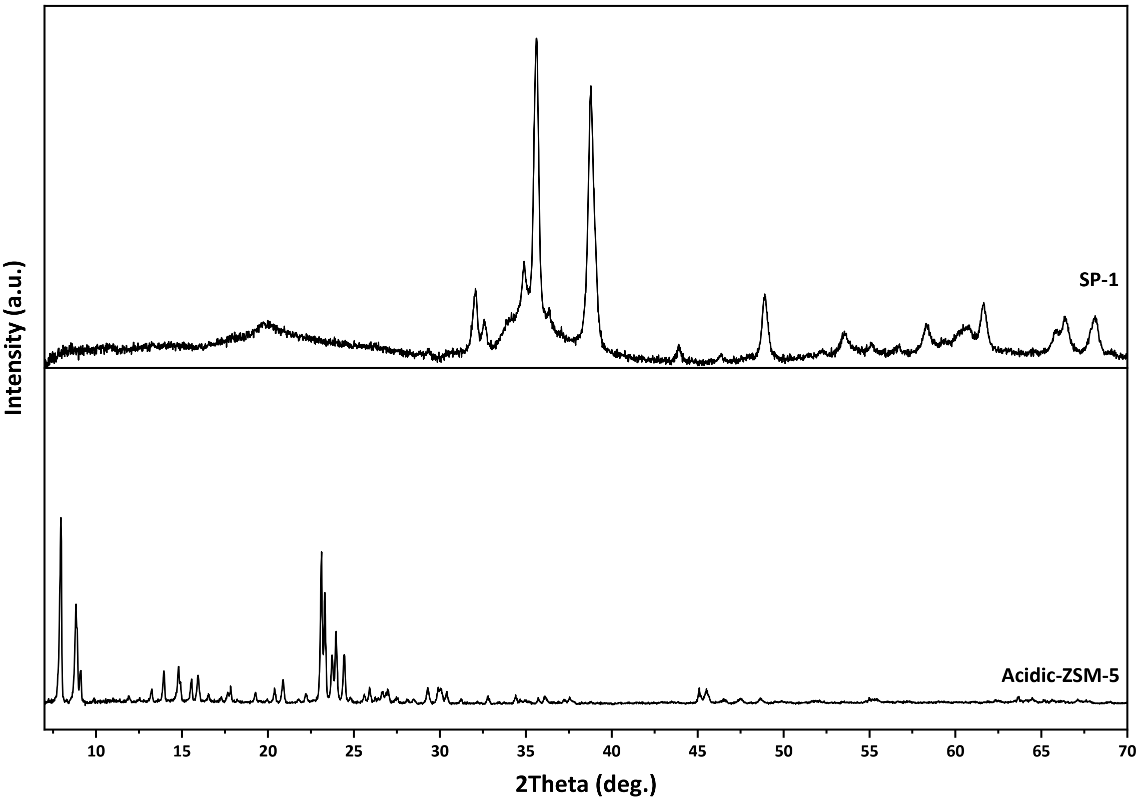

Figure S2. XRD patterns of SP-1 and Acidic-ZSM-5 powders, SP-1 in the present of metal nitrates.

As shown in Figure S3, the TGA profile consists of four regions in which each region is related to a specific weight loss. A total weight loss of 16.7% was occurred from room temperature to 1100 °C. All weight loss regions were shown at the reflection points. The first weight loss occurred at temperatures between 250 °C to 300 °C which can be attributed to desorption of inter-crystalline water. The weight loss occurred at the temperature region between 300 to 490 °C can be ascribed for decomposition of organic components such as TPAOH. The third weight loss at the temperature between 490 °C to 600 °C can be attributed to decarbonation and removing of CO_3^{2-} from the structure and also decomposition of NH_4F [49,57]. The final weight loss was occurred at 800 °C after which no weight loss can be observed.

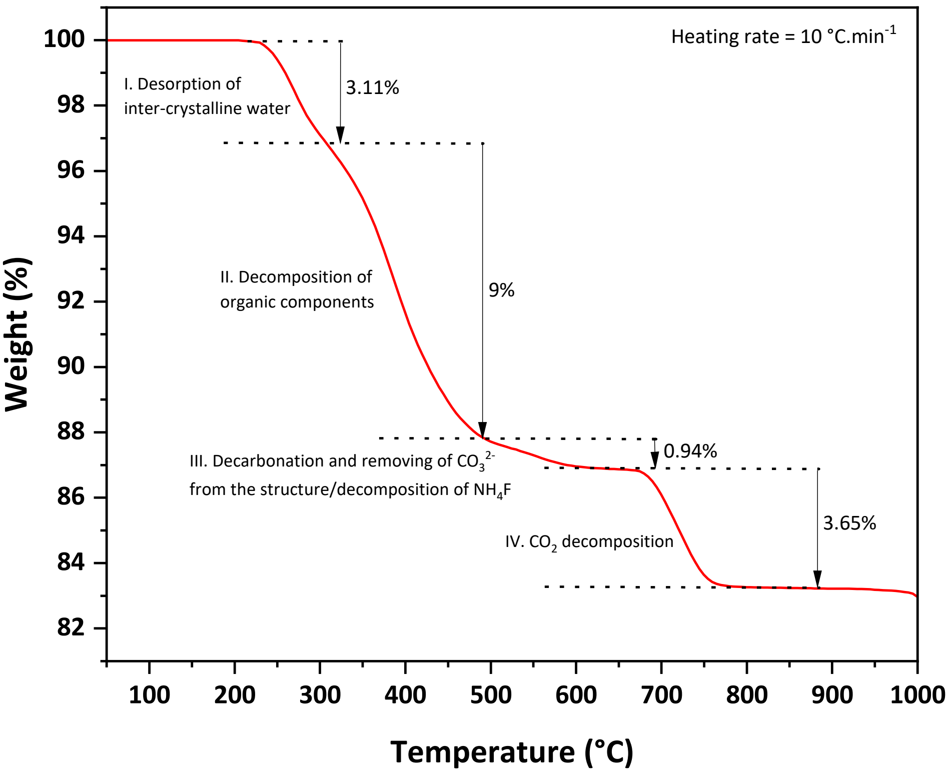

Figure S3. TGA curve corresponding to FSP-P.

The XRD patterns corresponded to FSP sample heat treated at different temperatures of 500, 600, 650, and 800 °C can be seen in Figure S4. As it can be seen there, zeolite was completely crystalized at temperature of 800 °C which is in accordance with the TGA results as why 800 °C was chosen as the calcination temperature for FSP sample.

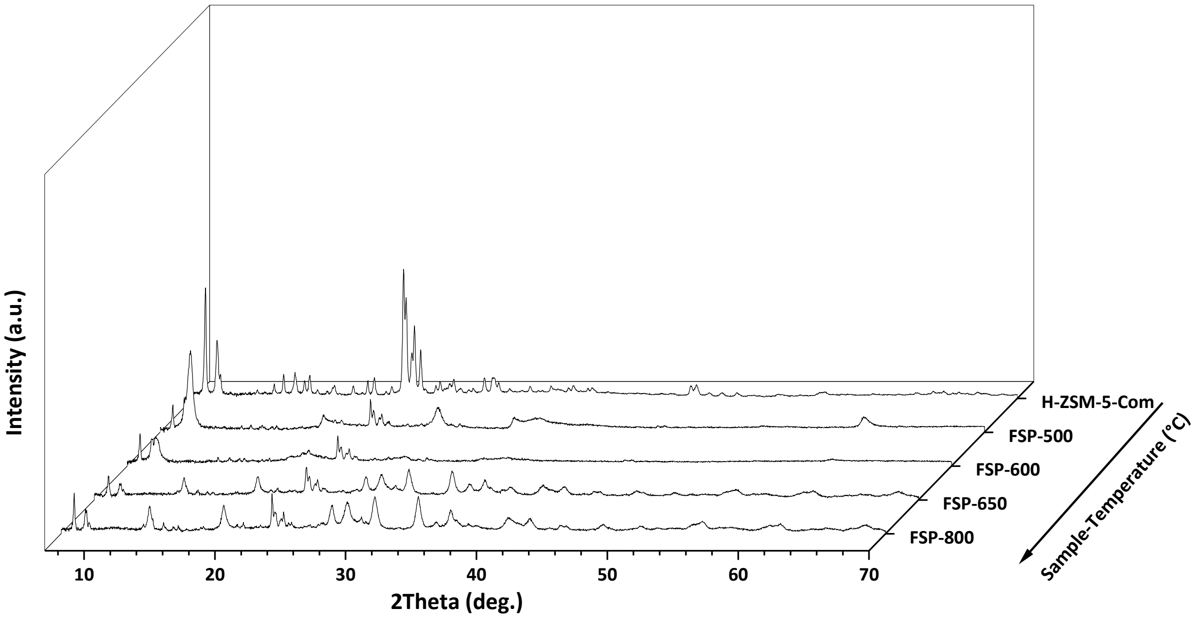

Figure S4. Waterfall XRD pattern of FSP calcined at different temperatures of 500 °C, 600 °C, 650 °C, and 800 °C. H-ZSM-5-Com is used as a reference sample.

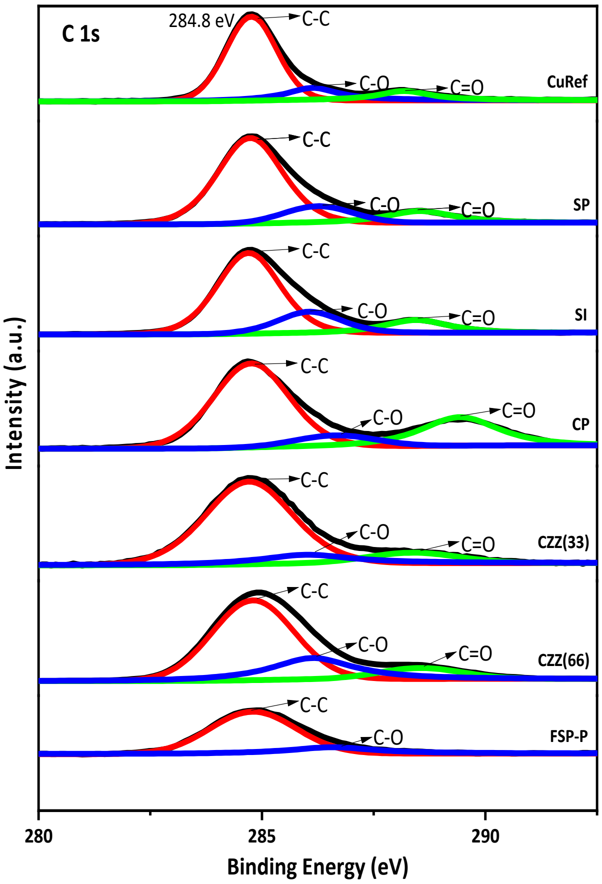

Figure S5. XPS of C 1s for all samples.

Figure S6 shows the XPS spectra of Cu 2p for fresh and spent catalysts including CP, CP-T, SI, SI-T, FSP-P, and FSP-P-T. As seen in Figure S6, the satellite feature peaks in Cu 2p spectra, which attributed to the Cu^{2+} species, were significantly decreased for all spent catalysts compared to the fresh catalysts, meaning that the Cu content in the catalysts was reduced to either Cu^{+} or Cu^{0}. It has been shown that Cu^{+} species play a more effective role than Cu^{0} in CO_2 hydrogenation to methanol [54,55]. As seen in Figure S6, the corresponding peaks of Cu $2p_{3/2}$ for all spent catalysts shifted to the lower binding energies which can be attributed to the presence of either metallic Cu (Cu^{0}) or Cu^{+} in the spent catalysts. Meaning that the spent catalysts were contributed in CO_2 conversion to methanol, especially through the Cu^{+} species. However, due to the small difference in binding energies between Cu^{+} and Cu^{0}, it is challenging to distinguish Cu^{+} from Cu^{0} in XPS spectra to calculate the quantity of each species [56].

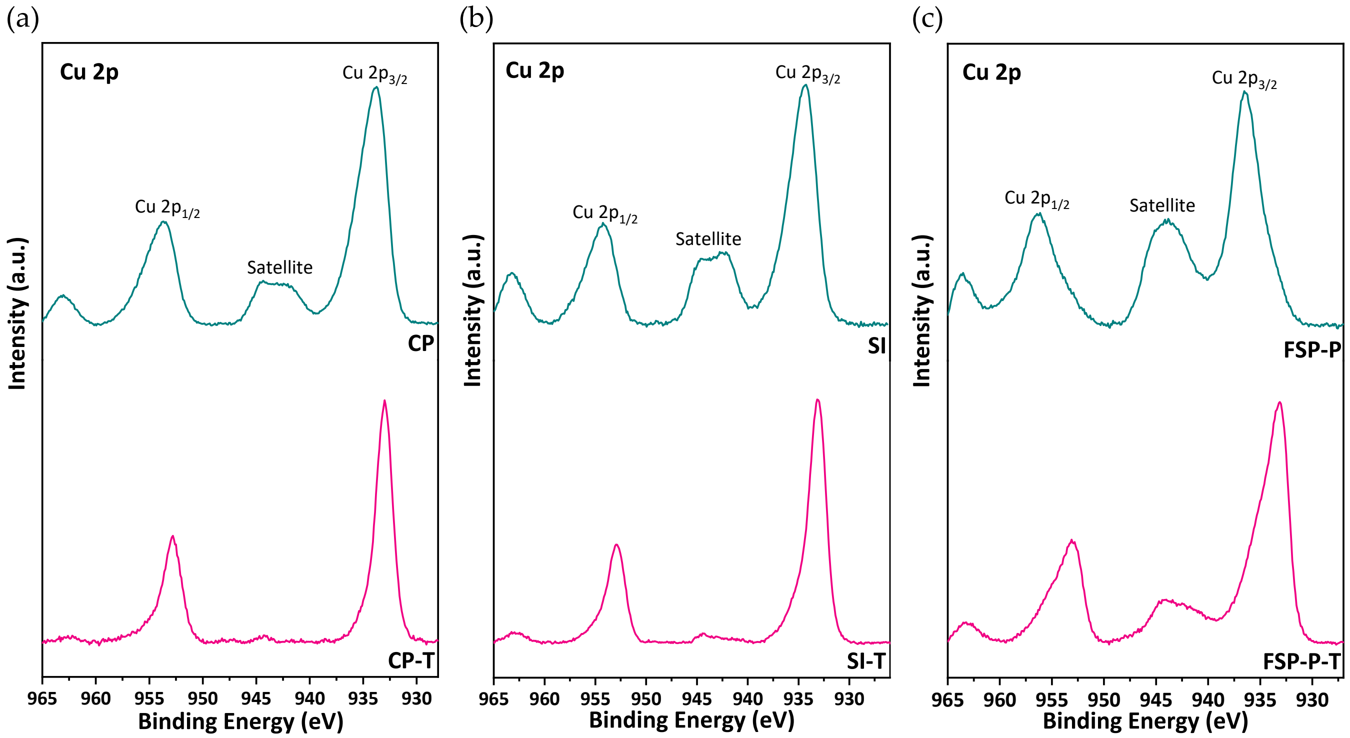

Figure S6. XPS spectra of Cu 2p for fresh and spent catalysts of (a) CP and CP-T, (b) SI and SI-T, and (c) FSP-P and FSP-P-T.

Figure S7 shows ammonia temperature-programmed desorption (NH_3-TPD) profiles for all catalysts including CP, SP, SI, CZZ(33), CZZ(66), and FSP-P. In order to better compare the acidity properties, the analysis was also performed for H-ZSM-5-P. As reported in literature [58], the NH_3-TPD profile plots can be divided into three zones of weak, medium, and strong acidities. These zones were marked in Figure S7 as I, II, and III for weak, medium, and strong acidity, respectively. The weak and medium acidity can be distinguished in the temperature ranges of 100-200 °C and 200-400 °C, respectively and the strong acidity can be found at 400-600 °C [58]. Pure zeolites with no metal typically exhibit two zones of I and II ascribed for weak and moderate acid sites, respectively [59]. As seen in Figure S7, the corresponding weak acidity peak at temperatures between 100-200 °C is high for H-ZSM-5-P compared to the samples in which Cu and Zn are present. CP which is avoid of zeolite exhibits a medium acidity without showing a weak or medium acidity peaks. SP and SI show a broad acidity peak of weak and medium together, however, SI shows the strong acidity at higher temperatures compared to SP. As reported elsewhere, by increasing the CuO active sites in zeolite structure, the catalyst shows higher strong acidity in NH_3-TPD [59]. As seen, the peak area of zone III for SP catalyst is more than SI, which can be attributed to the higher amount of CuO (33.33%wt.) in SP compared to SI with 16.67%wt. of CuO. The fibrous catalysts of CZZ(33) and CZZ(66) show low weak and medium acidity. A combination of weak and medium acidities can be seen for FSP-P which also a strong acidity peak can be found for this catalyst.

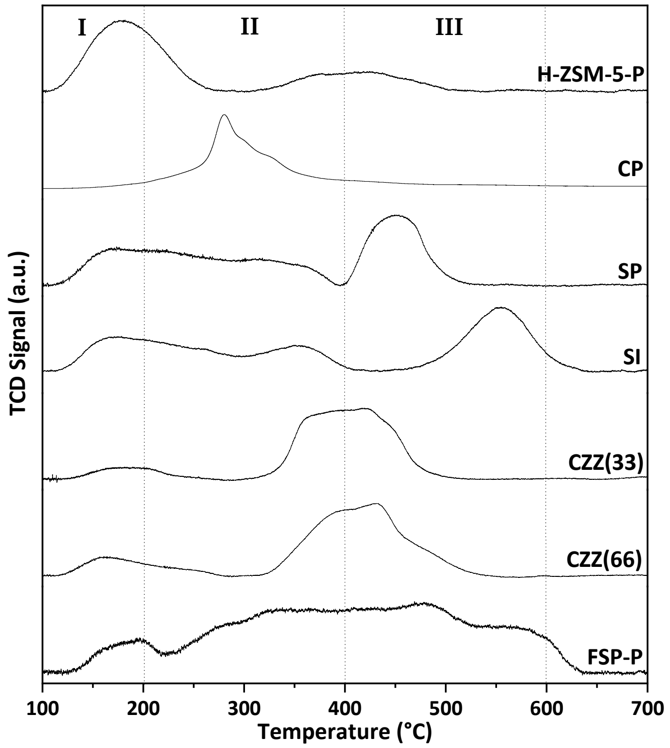

Figure S7. NH_3-TPD profile for all catalysts and H-ZSM-5-P for comparison.

Table S1. The Cu 2p XPS feature peaks, corresponding chemical shifts, and Cu^{2+} concentration.

Sample	Binding Energy (eV)		Chemical Shift (eV)*	I Cu $2p_{3/2}$ (a.u.)
	Cu $2p_{3/2}$	*Cu* $2p_{1/2}$		
**CuRef**	933.37	953.27	0	77556.24
**SP**	933.32	953.22	-0.05	7575.8
**SI**	934.34	954.24	+0.97	17175.24
**CP**	933.88	953.78	+0.51	25362.33
**CZZ(33)**	934.11	954.01	+0.74	16224.6
**CZZ(66)**	934.46	954.36	+1.09	23943.9
**FSP-P**	936.52	956.42	+3.15	12708.15

**Compared to the CuRef sample.*

Table S2. The Zn 2p XPS feature peaks and corresponding intensities.

Sample	Binding Energy (eV)		Chemical Shift (eV)*	I Zn $2p_{3/2}$ (a.u.)
	Zn $2p_{3/2}$	*Zn* $2p_{1/2}$		
**ZnRef**	1021.70	1044.70	0	318165.34
**SP**	1022.23	1045.23	+0.53	45533.39
**SI**	1022.57	1046.32	+0.87	39887.12
**CP**	1022.22	1045.79	+0.52	61183.62
**CZZ(33)**	1022.00	1045.87	+0.3	102937.20
**CZZ(66)**	1023.43	1046.28	+0.73	79345
**FSP-P**	1023.06	1046.06	+1.04	19557.23

**Compared to the ZnRef sample.*

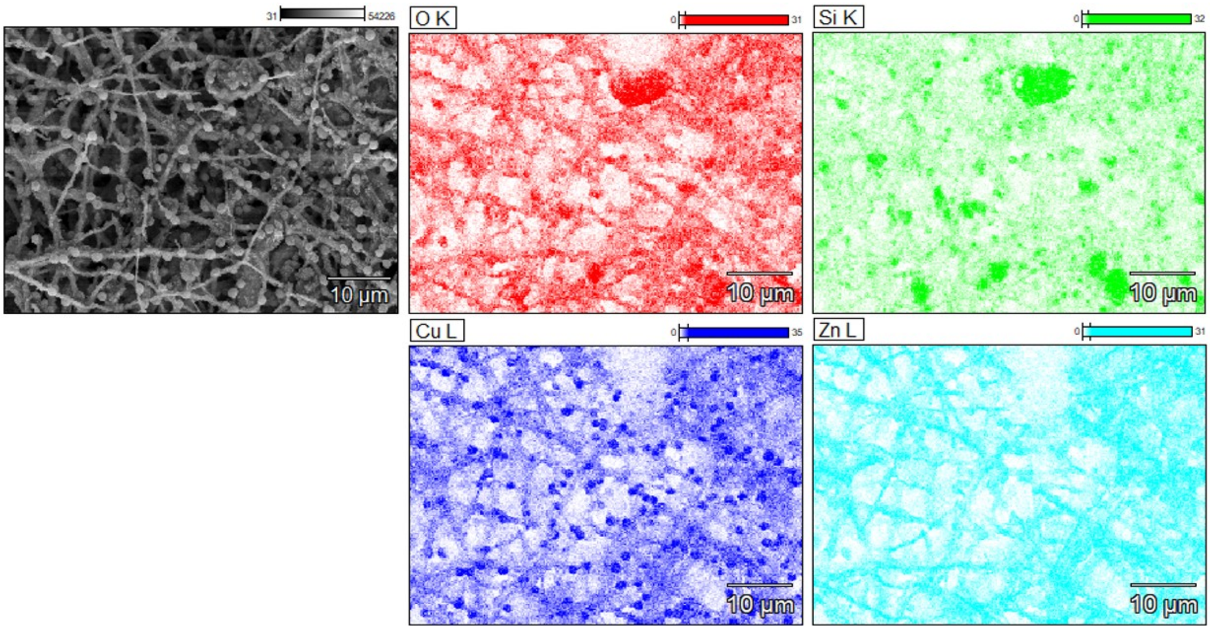

Figure S8. EDS analysis of CZZ(33) fibers after calcination.

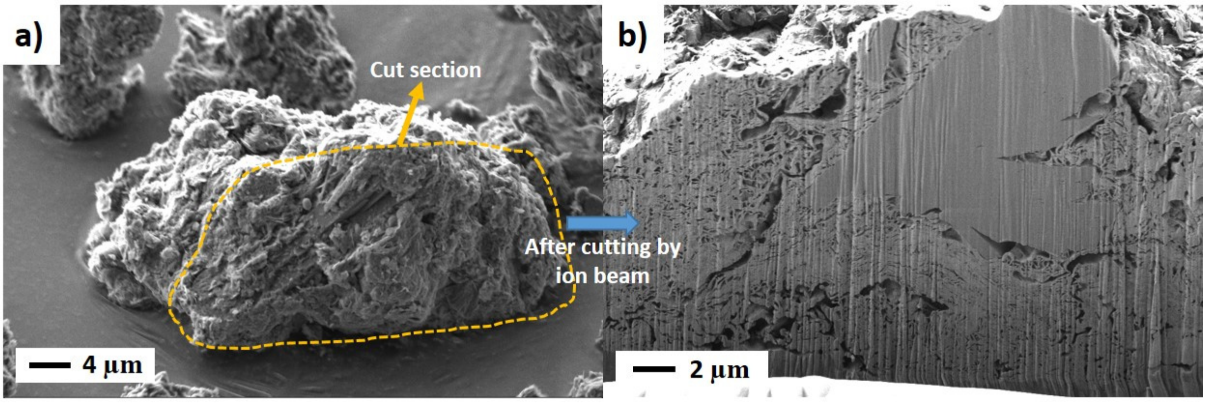

Figure S9. a) SEM image of FSP-P; the cut section shown, b) FIB-SEM of FSP-P after cutting by Gallium ion beam (EDS elemental mapping of this image is shown in Figure 11).

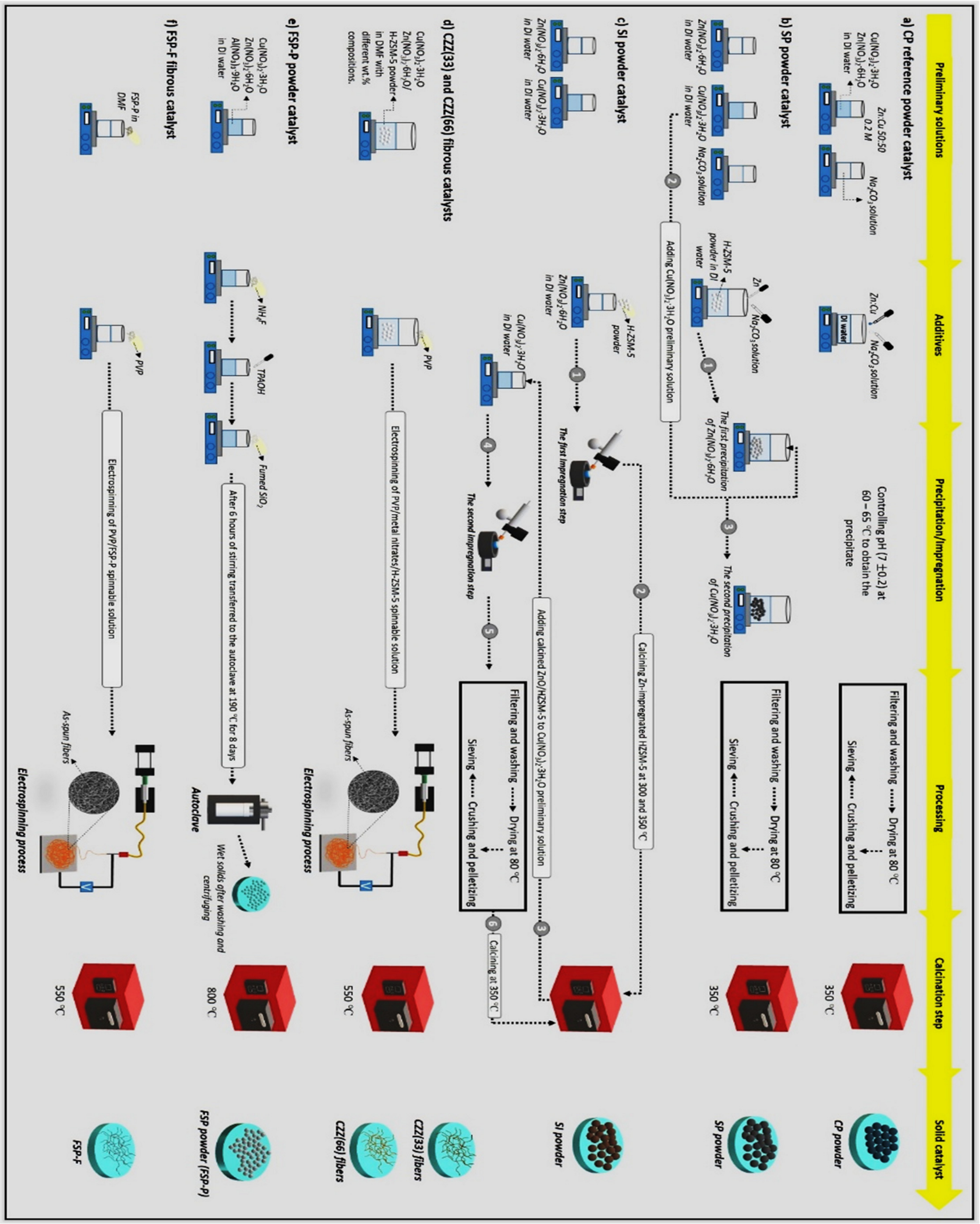

Figure S10. Synthesis and preparation procedures schematic of (a) CP reference powder catalyst, (b) SP powder catalyst, (c) SI powder catalyst, (d) CZZ(33) and CZZ(66) fibrous catalysts, and (e) FSP-P powder catalyst and (f) FSP-F fibrous catalyst.

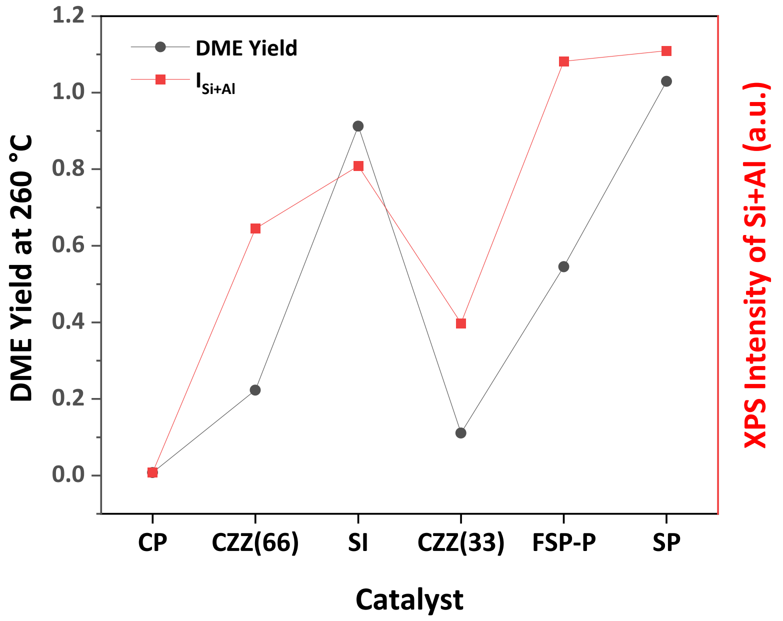

Figure S11. DME yield at 260 °C and XPS intensity of Si+Al as zeolite representatives.

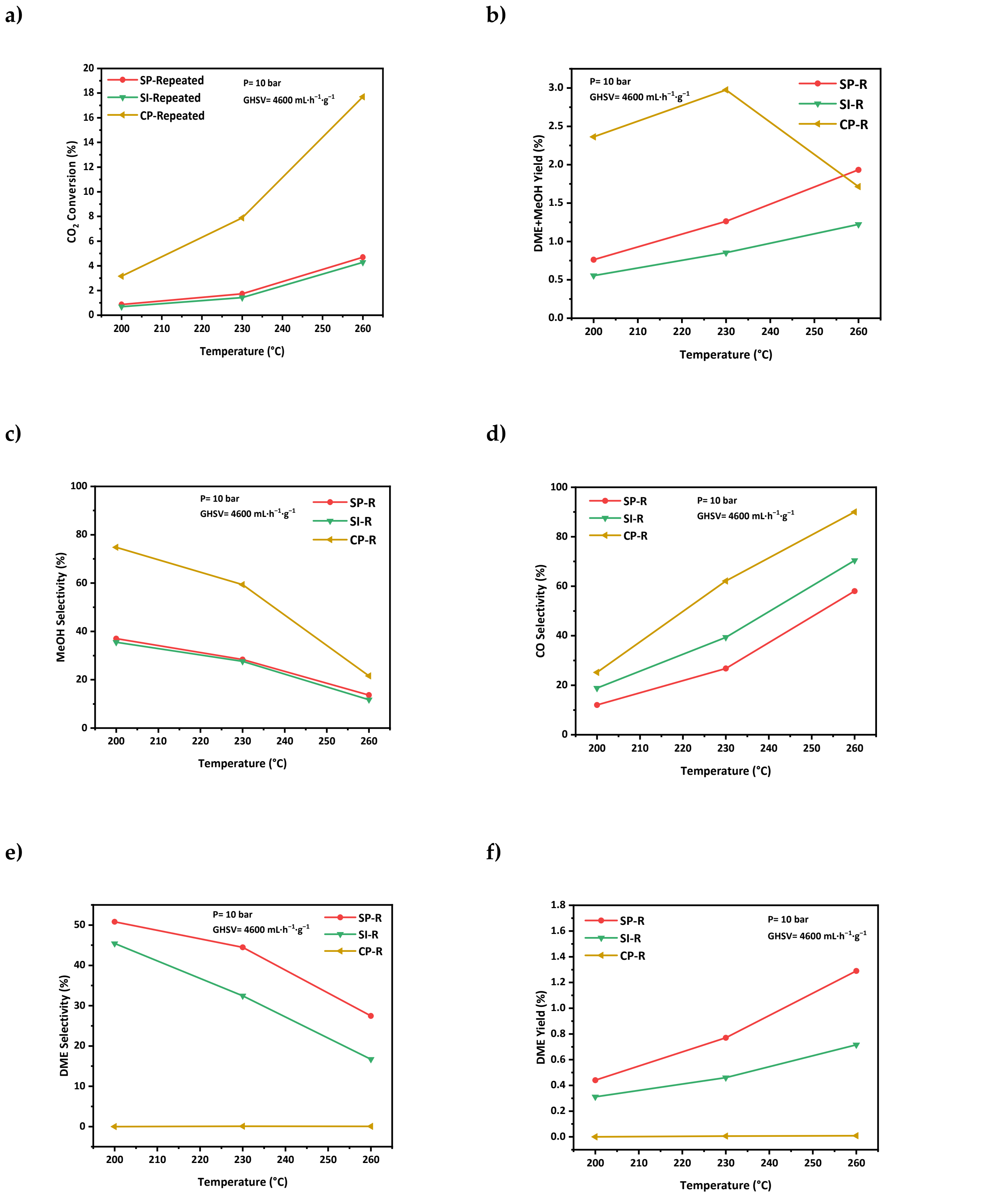

Figure S12. Reproducibility results for effect of reaction temperature on catalytic performance including a) CO_2 Conversion, b) DME+MeOH Yield, c) MeOH Selectivity, d) CO Selectivity, e) DME Selectivity, and f) DME-Yield at the pressure of 10 bar, the GHSV of 4600 mL.h^{-1}.g^{-1}, and H_2:CO_2=3:1.

a)

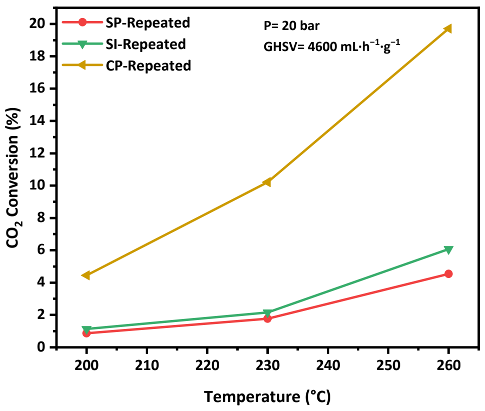

b)

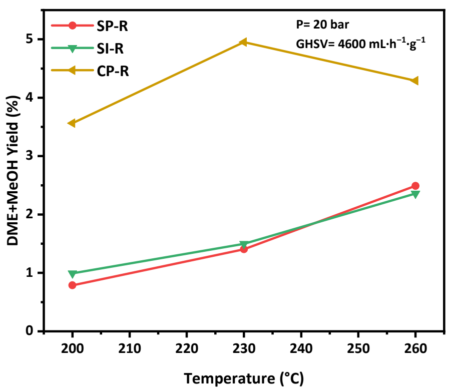

c)

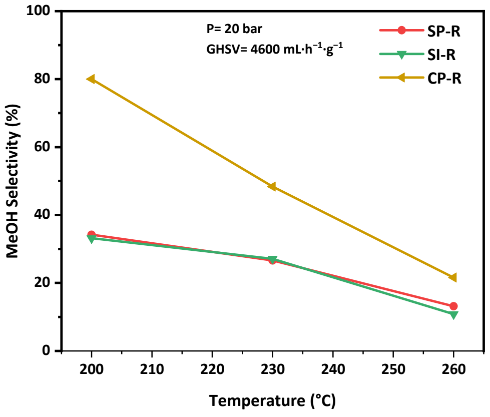

d)

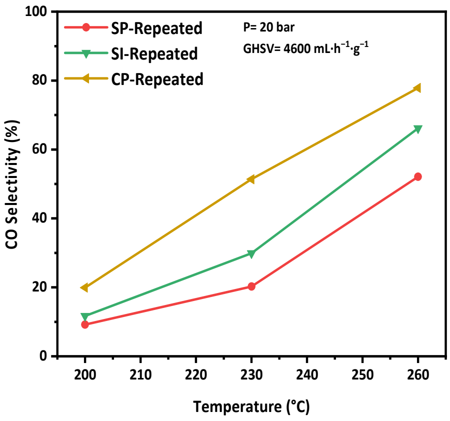

e)

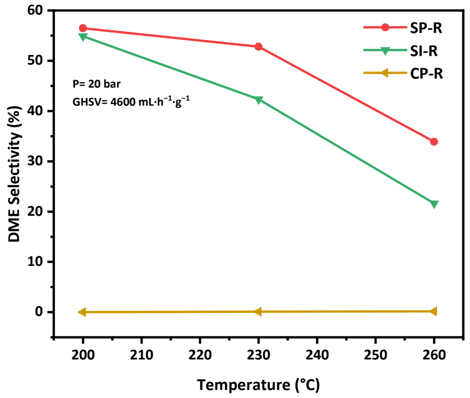

f)

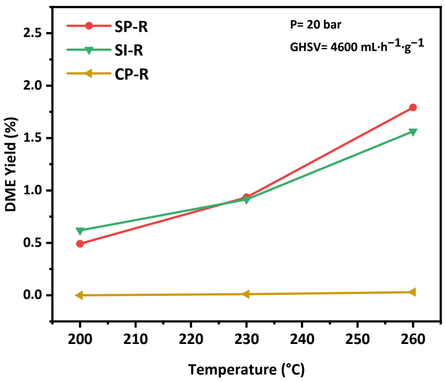

Figure S13. Reproducibility results for the effect of reaction temperature on catalytic performance including a) CO_2 Conversion, b) DME+MeOH Yield, c) MeOH Selectivity, d) CO Selectivity, e) DME Selectivity, and f) DME-Yield at the pressure of 20 bar, the GHSV of 4600 mL.h^{-1}.g^{-1}, and H_2:CO_2=3:1.

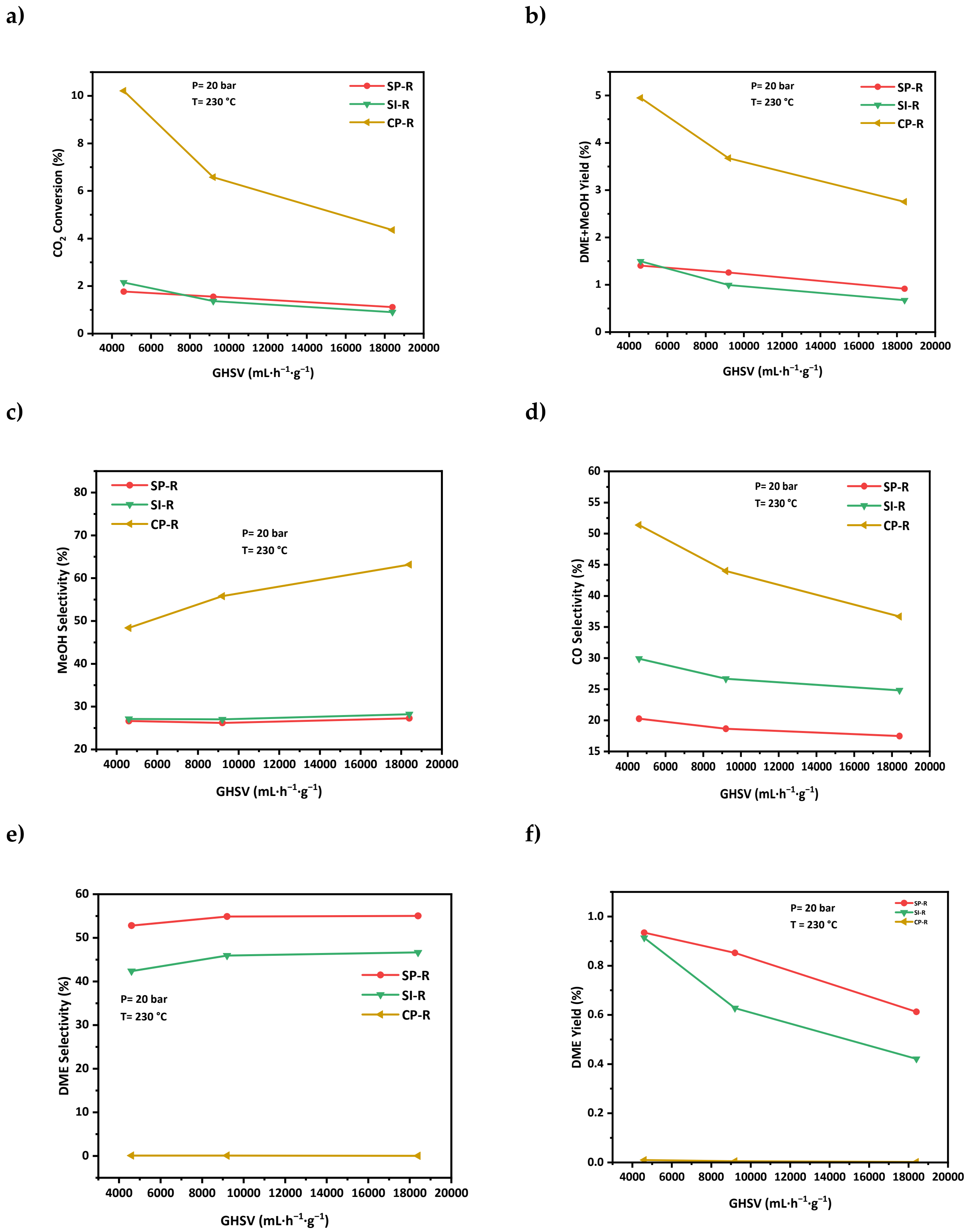

Figure S14. Reproducibility results for the effect of GHSV on the catalytic performance including a) CO_2 Conversion, b) DME+MeOH Yield, c) MeOH Selectivity, d) CO Selectivity, and e) DME Selectivity at the pressure of 20 bar, the temperature of 230 °C, and H_2:CO_2=3:1.

Table S3. Catalytic performance for all catalysts at different temperatures under 10 bar, the GHSV of 4600 $mL.h^{-1}.g^{-1}$, and H_2:CO_2=3:1

Catalyst	Preparation method	Composition (wt.%)	Operating condition			Catalytic performance				
			Temperature (°C)	*Pressure (bar)*	*GHSV ($mL.h^{-1}.g^{-1}$)*	X_{CO_2} (%)	*DME + MeOH yield (%)*	S_{MeOH} (%)	S_{CO} (%)	S_{DME} (%)
CP	Co-precipitation	50 CuO:50 ZnO	200	10	4600	3	2	69	30.74	0
CP-R*	Co-precipitation	50 CuO:50 ZnO	200	10	4600	3.15	2.36	74.82	25.17	0
CZZ(66)	Electrospinning	16.66 CuO:16.66 ZnO:66.66 ZSM-5	200	10	4600	1.23	1.13	90.40	8.5	1.1
CZZ(33)	Electrospinning	33.33 CuO:33.33 ZnO:33.33 ZSM-5	200	10	4600	1	0.83	83	16.1	0.57
SP-R*	Sequential-precipitation	33.33 CuO:33.33 ZnO:33.33 ZSM-5	200	10	4600	0.87	0.55	34.22	12	50.81
SI	Sequential-impregnation	16.66 CuO:16.66 ZnO:66.66 ZSM-5	200	10	4600	0.78	0.56	30.36	27.73	41.5
SP	Sequential-precipitation	33.33 CuO:33.33 ZnO:33.33 ZSM-5	200	10	4600	0.7	0.52	30.3	26.11	43.2
SI-R*	Sequential-impregnation	16.66 CuO:16.66 ZnO:66.66 ZSM-5	200	10	4600	0.68	0.55	35.5	18.83	45.42
FSP-F	Single-pot/electrospinning	16.66 CuO:16.66 ZnO:66.66 ZSM-5	200	10	4600	0.5	0.4	53.2	22.53	23.84
FSP-P	Single-pot	16.66 CuO:16.66 ZnO:66.66 ZSM-5	200	10	4600	0.47	0.35	48.3	24.53	26.75
CP-R*	Co-precipitation	50 CuO:50 ZnO	230	10	4600	7.9	3	59.37	62.11	0.07
CP	Co-precipitation	50 CuO:50 ZnO	230	10	4600	7.6	2.5	33	66.72	0.06
CZZ(66)	Electrospinning	16.66 CuO:16.66 ZnO:66.66 ZSM-5	230	10	4600	2	1.52	25.5	25.3	3.5
SI	Sequential-impregnation	16.66 CuO:16.66 ZnO:66.66 ZSM-5	230	10	4600	1.8	0.94	21.3	46.8	31
SP-R*	Sequential-precipitation	33.33 CuO:33.33 ZnO:33.33 ZSM-5	230	10	4600	1.73	1.26	28.4	26.8	44.5
CZZ(33)	Electrospinning	33.33 CuO:33.33 ZnO:33.33 ZSM-5	230	10	4600	1.7	1.1	64.6	32.75	1.8
SP	Sequential-precipitation	33.33 CuO:33.33 ZnO:33.33 ZSM-5	230	10	4600	1.63	0.9	21.9	43.45	33.8
SI-R*	Sequential-impregnation	16.66 CuO:16.66 ZnO:66.66 ZSM-5	230	10	4600	1.42	0.85	27.6	39.4	32.4
FSP-P	Single-pot	16.66 CuO:16.66 ZnO:66.66 ZSM-5	230	10	4600	1.21	0.65	30.45	45.2	23.46
FSP-F	Single-pot/electrospinning	16.66 CuO:16.66 ZnO:66.66 ZSM-5	230	10	4600	1	0.57	34.11	42.3	22.55
CP	Co-precipitation	50 CuO:50 ZnO	260	10	4600	18	1.56	19.75	90.75	0.04
CP-R*	Co-precipitation	50 CuO:50 ZnO	260	10	4600	17.7	1.7	21.6	90	0.05
SI	Sequential-impregnation	16.66 CuO:16.66 ZnO:66.66 ZSM-5	260	10	4600	6.2	1.6	12.38	72.83	14.72
SP	Sequential-precipitation	33.33 CuO:33.33 ZnO:33.33 ZSM-5	260	10	4600	5.4	1.71	13.31	67.11	19.1
SP-R*	Sequential-precipitation	33.33 CuO:33.33 ZnO:33.33 ZSM-5	260	10	4600	4.7	1.93	13.67	58	27.44
CZZ(33)	Electrospinning	33.33 CuO:33.33 ZnO:33.33 ZSM-5	260	10	4600	4.4	1.5	39.53	64.92	2.52
CZZ(66)	Electrospinning	16.66 CuO:16.66 ZnO:66.66 ZSM-5	260	10	4600	4.37	1.8	43.5	57.6	5.1
SI-R*	Sequential-impregnation	16.66 CuO:16.66 ZnO:66.66 ZSM-5	260	10	4600	4.28	1.22	11.73	70.43	16.7
FSP-P	Single-pot	16.66 CuO:16.66 ZnO:66.66 ZSM-5	260	10	4600	4	1	11.4	73.65	13.6
FSP-F	Single-pot/electrospinning	16.66 CuO:16.66 ZnO:66.66 ZSM-5	260	10	4600	3.4	0.9	12.7	72.1	13.5

**The repeated catalyst.*

Table S4. Catalytic performance for all catalysts at different temperatures under 20 bar, the GHSV of 4600 $mL.h^{-1}.g^{-1}$, and H_2:CO_2=3:1

Catalyst	Preparation method	Composition (wt.%)	Operating condition			Catalytic performance				
			Temperature (°C)	*Pressure (bar)*	*GHSV ($mL.h^{-1}.g^{-1}$)*	X_{CO_2} (%)	*DME + MeOH yield (%)*	S_{MeOH} (%)	S_{CO} (%)	S_{DME} (%)
CP-R*	Co-precipitation	50 CuO:50 ZnO	200	20	4600	4.45	3.56	80	19.92	0
CP	Co-precipitation	50 CuO:50 ZnO	200	20	4600	3.95	1.6	75.5	24.33	0
CZZ(66)	Electrospinning	16.66 CuO:16.66 ZnO:66.66 ZSM-5	200	20	4600	1.74	1.16	91.27	7.7	0.94
CZZ(33)	Electrospinning	33.33 CuO:33.33 ZnO:33.33 ZSM-5	200	20	4600	1.33	0.83	86.32	13	0.5
SI-R*	Sequential-impregnation	16.66 CuO:16.66 ZnO:66.66 ZSM-5	200	20	4600	1.13	0.99	33.16	11.73	54.9
SI	Sequential-impregnation	16.66 CuO:16.66 ZnO:66.66 ZSM-5	200	20	4600	1.07	0.83	29	21.8	48.9
SP	Sequential-precipitation	33.33 CuO:33.33 ZnO:33.33 ZSM-5	200	20	4600	0.97	0.76	28.3	20.9	50.4
FSP-F	Single-pot/electrospinning	16.66 CuO:16.66 ZnO:66.66 ZSM-5	200	20	4600	0.88	0.73	50.1	16.82	32.68
SP-R*	Sequential-precipitation	33.33 CuO:33.33 ZnO:33.33 ZSM-5	200	20	4600	0.87	0.8	34.2	9.2	56.5
FSP-P	Single-pot	16.66 CuO:16.66 ZnO:66.66 ZSM-5	200	20	4600	0.85	0.66	41.6	22.26	35.7
CP-R*	Co-precipitation	50 CuO:50 ZnO	230	20	4600	10.2	5	48.37	51.4	0.1
CP	Co-precipitation	50 CuO:50 ZnO	230	20	4600	9.6	4.26	44.24	55.32	0.09
CZZ(66)	Electrospinning	16.66 CuO:16.66 ZnO:66.66 ZSM-5	230	20	4600	3	2.3	75.9	20.16	3.3
CZZ(33)	Electrospinning	33.33 CuO:33.33 ZnO:33.33 ZSM-5	230	20	4600	2.5	1.85	72.32	25.3	1.7
SI	Sequential-impregnation	16.66 CuO:16.66 ZnO:66.66 ZSM-5	230	20	4600	2.44	1.5	21.72	37.43	40
SP	Sequential-precipitation	33.33 CuO:33.33 ZnO:33.33 ZSM-5	230	20	4600	2.23	1.46	22	33.47	43.7
Si-R*	Sequential-impregnation	16.66 CuO:16.66 ZnO:66.66 ZSM-5	230	20	4600	2.15	1.5	27.1	29.9	42.43
FSP-P	Single-pot	33.33 CuO:33.33 ZnO:33.33 ZSM-5	230	20	4600	2.04	1.2	27.9	40.52	30.75
SP-R*	Sequential-precipitation	16.66 CuO:16.66 ZnO:66.66 ZSM-5	230	20	4600	1.77	1.5	20.26	45.2	52.8
FSP-F	Single-pot/electrospinning	16.66 CuO:16.66 ZnO:66.66 ZSM-5	230	20	4600	1.74	1.1	35.66	42.3	31.2
CP-R*	Co-precipitation	50 CuO:50 ZnO	260	20	4600	19.7	4.3	21.6	77.9	0.16
CP	Co-precipitation	50 CuO:50 ZnO	260	20	4600	19	3.8	19.75	79.2	0.14
SI	Sequential-impregnation	16.66 CuO:16.66 ZnO:66.66 ZSM-5	260	20	4600	8.2	2.76	12.38	64.82	21.4
SP	Sequential-precipitation	33.33 CuO:33.33 ZnO:33.33 ZSM-5	260	20	4600	7.2	2.76	13.31	60.16	25.2

CZZ(33)	Electrospinning	33.33 CuO:33.33 ZnO:33.33 ZSM-5	260	20	4600	6.63	2.8	39.53	56.5	2.5
CZZ(66)	Electrospinning	16.66 CuO:16.66 ZnO:66.66 ZSM-5	260	20	4600	6.6	3.2	43.5	49.8	5.15
SI-R*	Sequential-impregnation	16.66 CuO:16.66 ZnO:66.66 ZSM-5	260	20	4600	6.07	2.36	10.8	66.2	21.7
FSP-P	Single-pot	16.66 CuO:16.66 ZnO:66.66 ZSM-5	260	20	4600	6.07	1.9	10.1	71.7	16.62
FSP-F	Single-pot/electrospinning	16.66 CuO:16.66 ZnO:66.66 ZSM-5	260	20	4600	5.1	1.7	11.2	69.8	17.1
SP-R	Sequential-precipitation	33.33 CuO:33.33 ZnO:33.33 ZSM-5	260	20	4600	4.5	2.5	13.1	52.13	33.9

* *The repeated catalyst.*

Table S5. Catalytic performance for all catalysts at different GHSVs under 20 bar, the temperature of 230 °C, and H2:CO2=3:1.

Catalyst	Preparation method	Composition (wt.%)	Operating condition			Catalytic performance				
			Temperature (°C)	*Pressure (bar)*	*GHSV (mL.h⁻¹.g⁻¹)*	X_{CO_2} (%)	*DME + MeOH yield (%)*	S_{MeOH} (%)	S_{CO} (%)	S_{DME} (%)
CP-R*	Co-precipitation	50 CuO:50 ZnO	230	20	4600	10.2	5	48.37	51.4	0.1
CP	Co-precipitation	50 CuO:50 ZnO	230	20	4600	9.6	4.26	44.24	55.32	0.09
CZZ(66)	Electrospinning	16.66 CuO:16.66 ZnO:66.66 ZSM-5	230	20	4600	3	2.3	75.9	20.16	3.3
CZZ(33)	Electrospinning	33.33 CuO:33.33 ZnO:33.33 ZSM-5	230	20	4600	2.5	1.85	72.32	25.3	1.7
Si-R*	Sequential-impregnation	16.66 CuO:16.66 ZnO:66.66 ZSM-5	230	20	4600	2.44	1.5	21.72	37.43	40
SI	Sequential-impregnation	16.66 CuO:16.66 ZnO:66.66 ZSM-5	230	20	4600	2.23	1.46	22	33.47	43.7
SP	Sequential-precipitation	33.33 CuO:33.33 ZnO:33.33 ZSM-5	230	20	4600	2.15	1.5	27.1	29.9	42.43
FSP-F	Single-pot/electrospinning	16.66 CuO:16.66 ZnO:66.66 ZSM-5	230	20	4600	2.04	1.2	27.9	40.52	30.75
SP-R*	Sequential-precipitation	33.33 CuO:33.33 ZnO:33.33 ZSM-5	230	20	4600	1.77	1.5	20.26	45.2	52.8
FSP-P	Single-pot	16.66 CuO:16.66 ZnO:66.66 ZSM-5	230	20	4600	1.74	1.1	35.66	42.3	31.2
CP-R*	Co-precipitation	50 CuO:50 ZnO	230	20	9200	6.58	3.7	55.8	44	0.08
CP	Co-precipitation	50 CuO:50 ZnO	230	20	9200	6.23	3.24	52	47.7	0.08
CZZ(66)	Electrospinning	16.66 CuO:16.66 ZnO:66.66 ZSM-5	230	20	9200	1.92	1.56	79	18.23	2.3
CZZ(33)	Electrospinning	33.33 CuO:33.33 ZnO:33.33 ZSM-5	230	20	9200	1.55	1.2	76.5	21.9	1.1
SI	Sequential-impregnation	16.66 CuO:16.66 ZnO:66.66 ZSM-5	230	20	9200	1.55	1.01	22.84	34	42.42
SP	Sequential-precipitation	33.33 CuO:33.33 ZnO:33.33 ZSM-5	230	20	9200	1.34	0.92	23.11	31	45.3
Si-R*	Sequential-impregnation	16.66 CuO:16.66 ZnO:66.66 ZSM-5	230	20	9200	1.36	0.99	27	26.7	45.9
FSP-P	Single-pot	33.33 CuO:33.33 ZnO:33.33 ZSM-5	230	20	9200	1.13	0.71	30.37	36.5	32.43
SP-R*	Sequential-precipitation	16.66 CuO:16.66 ZnO:66.66 ZSM-5	230	20	9200	1.55	1.26	26.2	18.7	54.9
FSP-F	Single-pot/electrospinning	16.66 CuO:16.66 ZnO:66.66 ZSM-5	230	20	9200	0.96	0.64	36	42.3	31
CP-R*	Co-precipitation	50 CuO:50 ZnO	230	20	18400	4.35	2.8	63	36.7	0.05
CP	Co-precipitation	50 CuO:50 ZnO	230	20	18400	4	3.24	60	39.7	0.05
SI	Sequential-impregnation	16.66 CuO:16.66 ZnO:66.66 ZSM-5	230	20	18400	1	0.7	24.5	31.65	43.4
SP	Sequential-precipitation	33.33 CuO:33.33 ZnO:33.33 ZSM-5	230	20	18400	0.85	0.6	25.13	29.85	44.5
CZZ(33)	Electrospinning	33.33 CuO:33.33 ZnO:33.33 ZSM-5	230	20	18400	1	0.8	80.6	18.3	0.8
CZZ(66)	Electrospinning	16.66 CuO:16.66 ZnO:66.66 ZSM-5	230	20	18400	2	1	80.6	17.5	1.5
SI-R*	Sequential-impregnation	16.66 CuO:16.66 ZnO:66.66 ZSM-5	230	20	18400	0.9	0.7	28.2	24.83	46.7
FSP-P	Single-pot	16.66 CuO:16.66 ZnO:66.66 ZSM-5	230	20	18400	0.64	0.4	35.5	33.9	30
FSP-F	Single-pot/electrospinning	16.66 CuO:16.66 ZnO:66.66 ZSM-5	230	20	18400	0.6	0.43	43.1	29.7	26.5
SP-R*	Sequential-precipitation	33.33 CuO:33.33 ZnO:33.33 ZSM-5	230	20	18400	1.1	0.9	27.3	17.5	55

* *The repeated catalyst.*

The CO_2 conversion rate was determined by a first-order rate constant (*k*) [60], and was calculated by the equation S1.

$$\boldsymbol{k = (\frac{-F_{CO_2}}{[CO_2]W}) \times \ln(1 - X_{CO_2})} \quad \text{(S1)}$$

Where F_{CO_2} is attributed to the mole of CO_2 in the reactor inlet gas stream, $[CO_2]$ indicates inlet molar concentration of CO_2, W is ascribed to weight of the catalyst in gram, and X_{CO_2} represents CO_2 conversion. Arrhenius equation (S2) was used to plot the Arrhenius plots [61]. Figure S15 represents Arrhenius plots for all catalysts as a function of 1000/T (K^{-1}).

$$\boldsymbol{lnk = lnA - \frac{E_a}{RT}} \quad \text{(S2)}$$

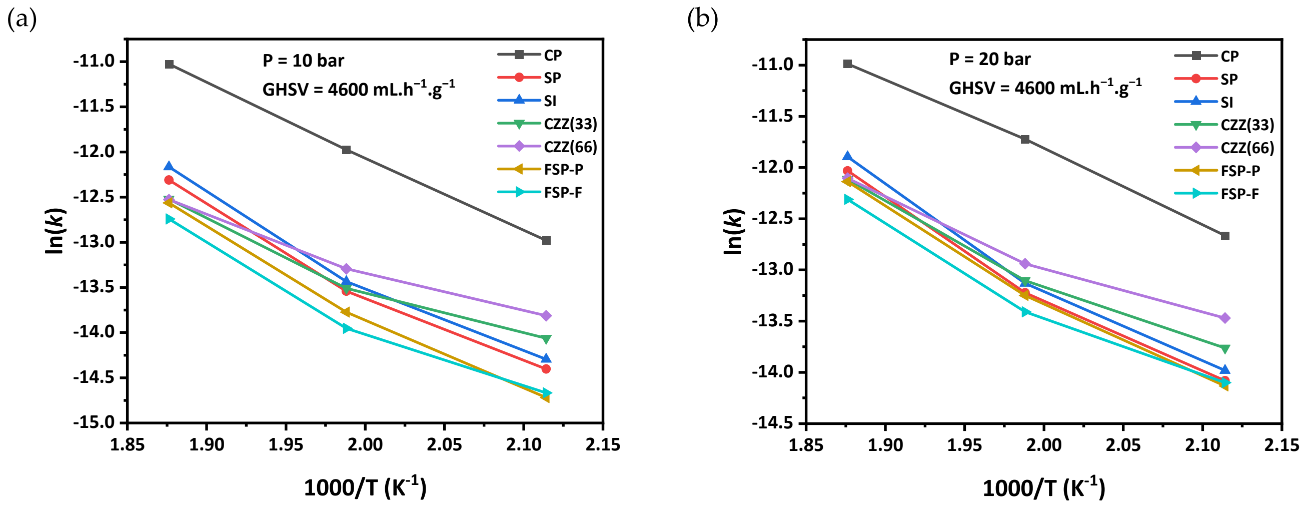

Figure S15. Arrhenius plots of all powder and fibrous catalysts including CP, SP, SI, CZZ(33), CZZ(66), FSP-P, and FSP-F (a) at the pressure of 10 bar and the GHSV of 4600 mL.h^{-1}.g^{-1}, and (b) at the pressure of 20 bar and the GHSV of 4600 mL.h^{-1}.g^{-1}.

Figure S16 represents the catalytic performance including CO_2 conversion, DME selectivity, and MeOH selectivity based on time on stream to test the stability of the catalysts. The catalyst's stability was monitored under the reaction condition of the pressure of 20 bar, the temperature of 230 °C for a time on stream of about 40 hours. As seen in Figure S16a, the CO_2 conversion remained almost constant for all catalysts except CP and CZZ(66). CP catalysts showed a decrease in CO_2 conversion after about 8 hours and remained partially constant during the next 20 hours, however, the CO_2 conversion decreased more in the stream between 28 to 40 hours. The CO_2 conversion for CZZ(66) showed a slight decrease after about 5 hours and remained constant over the whole stream. The DME selectivity was constant for SP and SI catalysts, however, a small increase can be seen for FSP-P and FSP-F catalysts in the time of stream of about 30 hours (Figure S16b). As seen in Figure S16c, the SP, SI, CZZ(66), and CZZ(33) catalysts showed steady trends for MeOH selectivity. The three catalysts of CP, FSP-F, and FSP-P exhibited an increase in MeOH selectivity in the last about 10-hour stream.

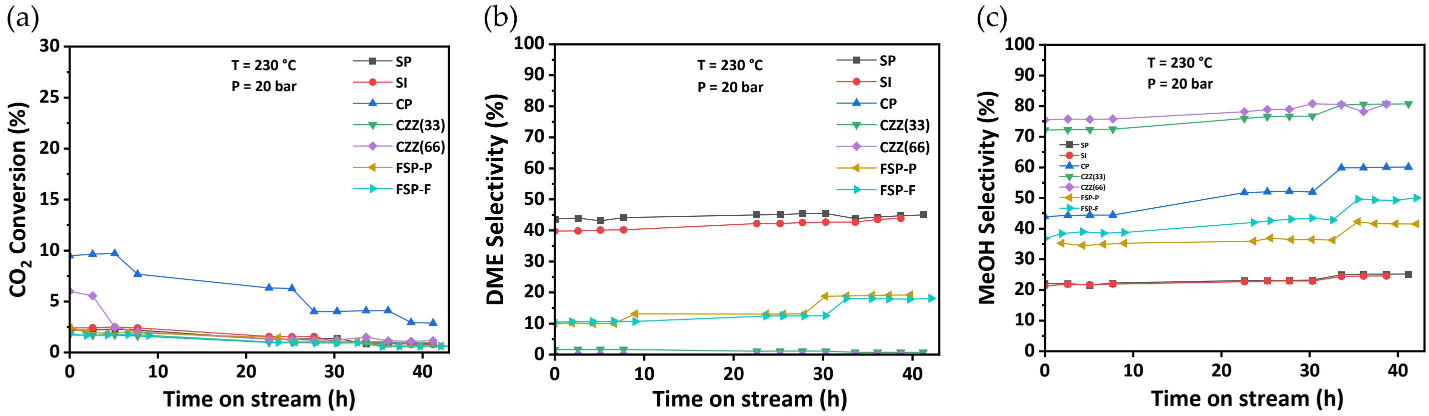

Figure S16. Stability of the catalysts as time on stream plots for (a) CO_2 conversion, (b) DME Selectivity, and (c) MeOH selectivity (reaction condition: GHSVs: 4600 mL.h^{-1}.g^{-1}, 9200 mL.h^{-1}.g^{-1}, and 18400 mL.h^{-1}.g^{-1}, P = 20 bar, T = 230 °C, and 250 mg of catalyst).

Figure S17 represents the SEM images of fresh and spent catalysts including SP, SP-T, SI, SI-T, FSP-P, and FSP-P-T. As seen in all images the morphology of the catalysts remained stabled after the reaction condition for spent catalysts and no significant change can be observed for spent catalysts.

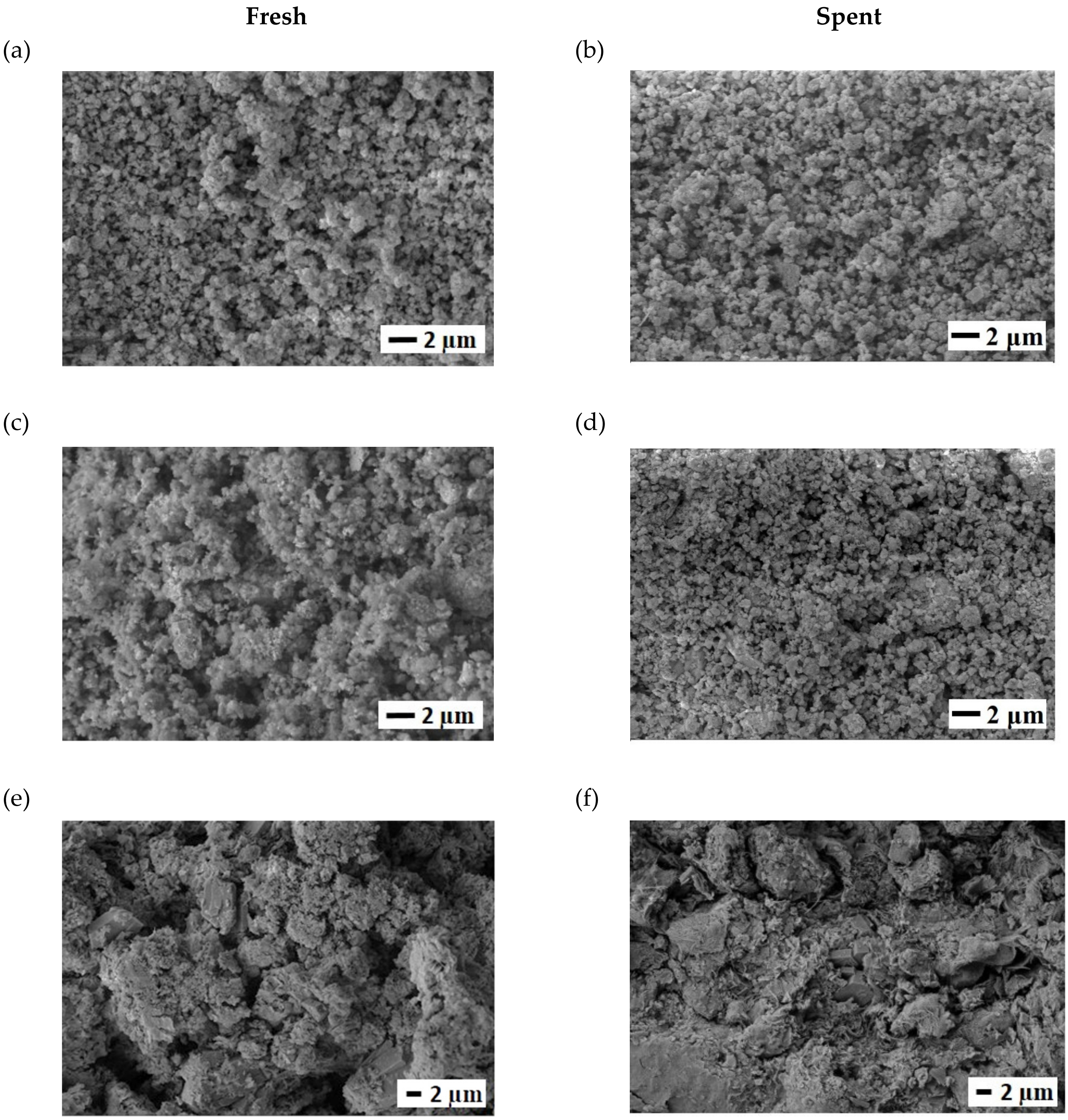

Figure S17. SEM images of fresh and spent catalysts of a) SP, b) SP-T, c) SI, d) SI-T, e) FSP-P, and f) FSP-P-T.
